# Dynamic changes in chromatin accessibility, altered adipogenic gene expression, and total versus de novo fatty acid synthesis in subcutaneous adipose stem cells of normal-weight polycystic ovary syndrome (PCOS) women during adipogenesis: evidence of cellular programming

**DOI:** 10.1186/s13148-020-00970-x

**Published:** 2020-11-23

**Authors:** Karen L. Leung, Smriti Sanchita, Catherine T. Pham, Brett A. Davis, Mariam Okhovat, Xiangming Ding, Phillip Dumesic, Tristan R. Grogan, Kevin J. Williams, Marco Morselli, Feiyang Ma, Lucia Carbone, Xinmin Li, Matteo Pellegrini, Daniel A. Dumesic, Gregorio D. Chazenbalk

**Affiliations:** 1 Department of Obstetrics and Gynecology, David Geffen School of Medicine at UCLA, Los Angeles, CA 90095 USA; 2 Department of Medicine, Knight Cardiovascular Institute, Oregon Health and Sciences University, Portland, OR 97239 USA; 3 Technology Center for Genomics and Bioinformatics, Department of Medicine, David Geffen School of Medicine at UCLA, Los Angeles, CA 90095 USA; 4 Dana-Farber Cancer Institute, Boston, MA 02115 USA; 5 Department of Medicine Statistics Core, David Geffen School of Medicine at UCLA, Los Angeles, CA 90095 USA; 6 UCLA Lipidomics Lab, Department of Biological Chemistry, University of California Los Angeles, Los Angeles, CA 90095 USA; 7 Department of Molecular, Cell, and Developmental Biology, University of California Los Angeles, Los Angeles, CA 90095 USA; 8 Department of Molecular and Medical Genetics, Oregon Health and Sciences University, Portland, OR 97239 USA; 9 Department of Medical Information and Clinical Epidemiology, Oregon Health and Sciences University, Portland, OR 97239 USA; 10 Division of Genetics, Oregon National Primate Research Center, Beaverton, OR 97006 USA

**Keywords:** Cellular programming, Adipogenesis, Adipose stem cells, Polycystic ovary syndrome, Fat storage, Chromatin accessibility, Transcriptional factors, Gene expression, Total content, De novo synthesis of fatty acids

## Abstract

**Background:**

Normal-weight polycystic ovary syndrome (PCOS) women exhibit adipose resistance in vivo accompanied by enhanced subcutaneous (SC) abdominal adipose stem cell (ASC) development to adipocytes with accelerated lipid accumulation per cell in vitro. The present study examines chromatin accessibility, RNA expression and fatty acid (FA) synthesis during SC abdominal ASC differentiation into adipocytes in vitro of normal-weight PCOS versus age- and body mass index-matched normoandrogenic ovulatory (control) women to study epigenetic/genetic characteristics as well as functional alterations of PCOS and control ASCs during adipogenesis.

**Results:**

SC abdominal ASCs from PCOS women versus controls exhibited dynamic chromatin accessibility during adipogenesis, from significantly less chromatin accessibility at day 0 to greater chromatin accessibility by day 12, with enrichment of binding motifs for transcription factors (TFs) of the AP-1 subfamily at days 0, 3, and 12. In PCOS versus control cells, expression of genes governing adipocyte differentiation (*PPARγ*, *CEBPα*, *AGPAT2*) and function (*ADIPOQ*, *FABP4*, *LPL*, *PLIN1*, *SLC2A4*) was increased two–sixfold at days 3, 7, and 12, while that involving Wnt signaling (*FZD1*, *SFRP1*, and *WNT10B)* was decreased. Differential gene expression in PCOS cells at these time points involved triacylglycerol synthesis, lipid oxidation, free fatty acid beta-oxidation, and oxidative phosphorylation of the TCA cycle, with *TGFB1* as a significant upstream regulator. There was a broad correspondence between increased chromatin accessibility and increased RNA expression of those 12 genes involved in adipocyte differentiation and function, Wnt signaling, as well as genes involved in the triacylglycerol synthesis functional group at day 12 of adipogenesis. Total content and de novo synthesis of myristic (C14:0), palmitic (C16:0), palmitoleic (C16:1), and oleic (C18:1) acid increased from day 7 to day 12 in all cells, with total content and de novo synthesis of FAs significantly greater in PCOS than controls cells at day 12.

**Conclusions:**

In normal-weight PCOS women, dynamic chromatin remodeling of SC abdominal ASCs during adipogenesis may enhance adipogenic gene expression as a programmed mechanism to promote greater fat storage.

## Background

As the most common reproductive-metabolic disorder of reproductive-aged women, polycystic ovary syndrome (PCOS) is characterized by hyperandrogenism, menstrual irregularity, and polycystic ovarian morphology [1]. Most women with PCOS also have insulin resistance that underlies metabolic dysfunction and accompanies increased abdominal body fat over a wide range of body mass index (BMI) [2–5]. Teleologically, PCOS has a heritable component that interacts with the endocrine-metabolic environment to exert both acute and programmed effects on its phenotype [6, 7]. In support of this concept, adult female monkeys with natural hyperandrogenemia have comparable PCOS-like traits, suggesting an evolutionary origin that originally favored PCOS in hunter-gatherers [8], when food deprivation during pregnancy programmed greater fat storage in the fetus to meet the increased metabolic demands of reproduction in later life [9].

Adipocytes normally accumulate lipid and utilize their energy stores during reduced caloric intake to maintain energy homeostasis [10, 11]. In subcutaneous (SC) abdominal adipocytes of PCOS women, however, hyperandrogenemia alters SC fat storage capacity through changes in its lipid formation (lipogenesis) and breakdown (lipolysis) in mature adipocytes and its formation of new adipocytes via adipogenesis, whereby multipotent adipose stem cells (ASCs) undergo commitment to preadipocytes and differentiate into newly-formed adipocytes [12–14]. In vitro studies have shown that androgen contributes to diminished insulin-stimulated glucose uptake and inhibition of catecholamine-stimulated lipolysis in this adipose depot [12, 15–17], with comparable findings in similar adipose of women with PCOS [18–20].

SC fat storage also is developmentally programmed [21], as evidenced by similarly altered adipose function in PCOS-like prenatally testosterone (T)-treated adult rhesus monkeys and sheep with increased visceral adiposity and insulin resistance [6, 8]. Recently, chromatin immunoprecipitation (ChIP) studies of two-year-old adult sheep ovaries also found a relationship between prenatal T-induced epigenetic changes with up- and down-regulated genes [22]. Moreover, differential methylation patterns accompanying expression of candidate adipogenic genes such as *PPARγ *have been observed in SC abdominal adipose of PCOS women compared to controls [23]. Modification of the *PPARγ *loci, alongside *CEBPα* [24], suggests that critical genes governing SC abdominal adipogenesis may be altered in PCOS through differential chromatin accessibility and/or transcriptional regulation that underlie functional differences in PCOS adipocytes.

In support of programmed effects on adipose function in PCOS, we previously have shown that SC abdominal ASCs from eight normal-weight PCOS by NIH criteria versus eight age- and BMI-matched control women cultured without androgen exhibit increased ASC commitment to preadipocytes that negatively correlates with circulating fasting glucose levels, while enhanced lipid accumulation in newly, in vitro-formed adipocytes positively correlates with circulating androgen levels, implying a cellular programmed mechanism to maintain glucose-insulin homeostasis when fat accretion is accelerated [25]. Importantly, ASCs from a subset of these normal-weight PCOS women showed enhanced lipid accumulation during adipogenesis in vitro in combination with overexpression of both *PPARγ* and *CEBPα*, (Additional file 1: Supplemental Fig. 1), as master regulator genes of adipogenesis required for adipocyte differentiation and maintenance [24, 26, 27], suggesting specific genetic/epigenetic ASC characteristics in these PCOS women that may contribute to overexpression of these critical adipogenic genes. Therefore, the present pilot study examined whether *PPARγ* and *CEBPα* overexpression combined with enhanced lipid accumulation during adipogenesis in vitro in this subgroup of PCOS ASCs is accompanied by changes in chromatin accessibility and/or transcriptional regulation compared to control stem cells, and if so, whether functional differences also exist in the total content and de novo synthesis of fatty acids in the newly-formed adipocytes derived from those ASCs.

**Fig. 1:**
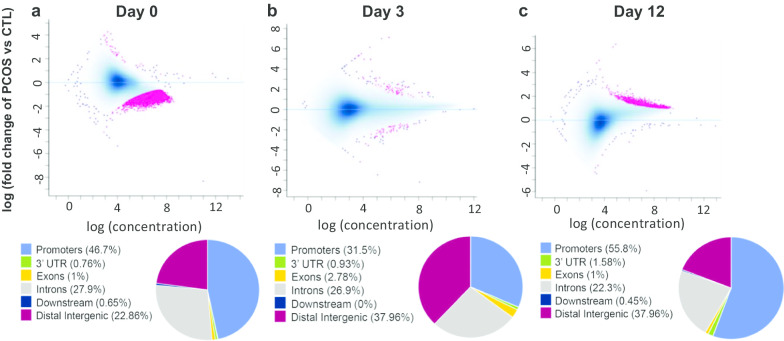
Scatter plot of pairwise DNA accessibility comparisons between control and PCOS samples at **a** day 0, **b** day 3 and **c** day 12. Each data point represent a genomic region, and those with significant accessibility difference (FDR < 0.05) between PCOS and control are highlighted in pink. Regions located above the blue horizontal line [log (fold change = 0)] have higher accessibility in PCOS, while those located below the line have lower accessibility. Pie charts below each plot display percentage overlap between significant differentially accessible regions and annotated genes

## Results

### Patient characteristics

Three normal-weight women with PCOS and three age- and BMI-matched normoandrogenic ovulatory (control) women (all women, 19–33 years; 19.5–23.9 kg/m^2^) who participated in our NIH study of adipogenic dysfunction in PCOS were selected from a cohort of eight PCOS women and their age- and BMI-matched controls. Selection of these three PCOS women was based upon their previously determined stem cell characteristics of enhanced lipid accumulation during adipogenesis in vitro [25] with *PPARγ* and *CEBPα* overexpression (Additional file 1: Supplemental Fig. 1). Women with PCOS were diagnosed by 1990 NIH criteria, while control women had normal menstrual cycles at 21- to 35-day intervals and a luteal phase progesterone (P4) level without hirsutism, acne or alopecia, as previously described [1, 5]. Serum total and free *T* levels were significantly higher in PCOS than controls (*p* < 0.030, *p* < 0.002, respectively), while serum A4, DHT, DHEAS, and LH levels were comparable between the two female groups. Insulin sensitivity, as determined by intravenous glucose tolerance testing, was in the low-normal range in the PCOS women, without female-type differences in fasting levels of plasma glucose and serum insulin or adipose-IR.

### ASC chromatin accessibility

To determine the degree of separation between female-type and time points, unsupervised principal component analysis of ATAC-seq data was based on read densities in consensus peak regions demonstrated grouping of samples primarily consistent with time-point and broadly with female type based on plots of the first two principal components (Additional file 2: Supplemental Fig. 2A). Of note, PCOS samples appeared distinctly separated from control samples at day 0, while the separation among day 3 and day 12 samples was less defined, suggesting higher similarity among these samples.

**Fig. 2:**
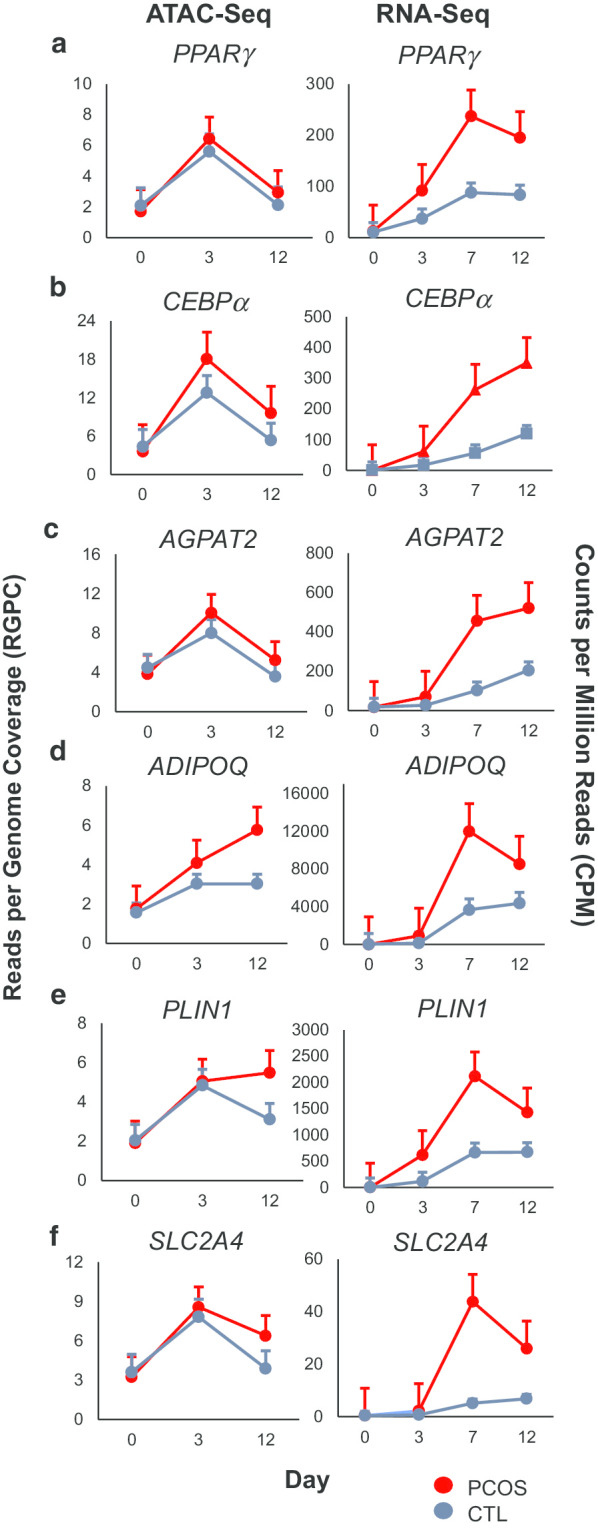
Chromatin accessibility and gene expression patterns of selected genes involved in adipogenesis and adipocyte function throughout adipogenesis. Genes actively involved in (**a**–**c**) adipogenesis and (**d**–**f**) adipocyte function are shown. Left panel represents the chromatin accessibility of associated gene regions at days 0, 3, and 12 of adipogenesis. Right panel represents RNA expression levels of each gene at days 0, 3, 7, and 12 of adipogenesis. Values are expressed as mean ± SEM of 3 age and BMI pair-matched normal-weight PCOS and control women

To investigate potential changes in gene regulation at the level of chromatin accessibility across time-points within each female-type and between female types, ATAC-seq read density within 3 kb of all transcription start sites (TSS) of genes was assessed. When assessing absolute chromatin accessibility of each female-type, highest ATAC-seq read density was found at day 3 compared to day 0 or day 12 for both female types, indicating increased accessibility of gene promoters to transcription factors and other regulators of gene expression at day 3 for both groups (Additional file 2: Supplemental Fig. 3). Of note, when gene chromatin accessibility was compared in PCOS relative to control samples, there was a switch in TSS chromatin accessibility of PCOS samples from day 0 to day 12 of adipogenesis, where TSS regions appeared overall less accessible in PCOS compared to controls at day 0, were comparable to controls at day 3, but became globally more accessible than controls by day 12.

**Fig. 3:**
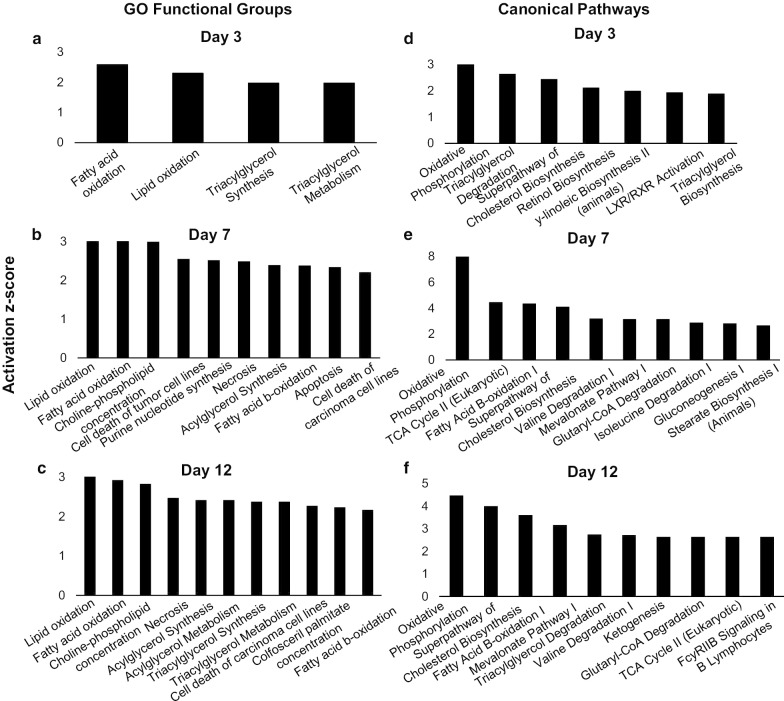
Functional group categories and canonical pathways in ASCs at days 3, 7, and 12 of adipogenesis. **a**–**c** Functional group categories and **d**–**f** canonical pathways of differentially expressed genes between newly-formed adipocytes from SC abdominal ASCs of normal-weight PCOS and control women at **a**, **d** day 3, **b**, **e** day 7, and **c**, **f** day 12, as generated by Ingenuity Pathway Analysis

This shift in chromatin accessibility was further validated after identifying statistically significant regions of differential accessibility between female types across time. At day 0, nearly all statistically significant differentially accessible regions (10,250 out of 10,287; 99.6%) were less accessible in PCOS compared to controls (Fig. 1a). At day 3, only 108 differential accessibility regions were found, and less than half (43 or 39.8%) showed lower accessibility in PCOS compared to controls (Fig. 1b), whereas at day 12 and converse to patterns observed at day 0, almost all 1201 significant differentially accessible regions identified (*N* = 1197; 99.6%) displayed higher accessibility in PCOS compared to control cells (Fig. 1c). Furthermore, at both days 0 and 12, a large portion of the significant differentially accessible regions overlapped promoters (39.57% at day 0 and 50.29% at day 12), consistent with the shift in TSS chromatin accessibility between PCOS and control cells across time (Additional file 2: Supplemental Fig. 3). These quantitative data further support the shift in chromatin accessibility of PCOS cells from less accessible than controls at day 0 to more accessible than controls by day 12, with day 3 as the transition time point.

Among the regions of differential accessibility identified in pairwise comparisons of PCOS versus control cells, most regions were only found at one of the time points, indicating time-specific differences. In contrast, a smaller, yet noteworthy, subset of regions was shared across two or three time points (Additional file 2: Supplemental Fig. 4). Interestingly, day 0 and 12 chromatin accessibility comparisons between female types had the highest number of shared regions (344 regions), all of which were less accessible in PCOS samples (relative to control) at day 0, but were more accessible in PCOS samples at day 12. The number of differentially accessible regions for each sample at each time point and detailed information regarding each region is reported in Additional file 3: Supplemental Table 1.

**Fig. 4:**
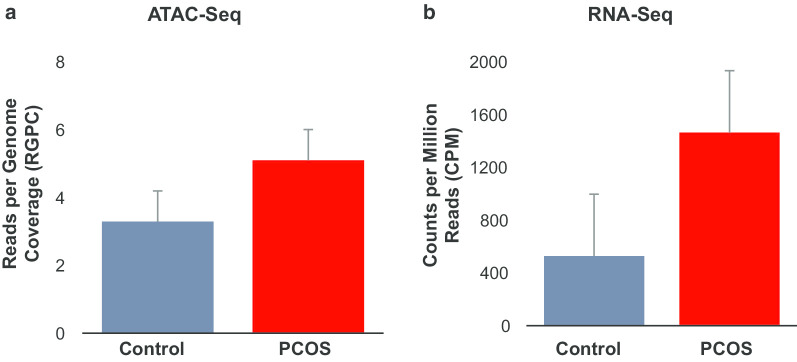
**a** Chromatin accessibility and **b** RNA expression of genes in the triacylglycerol synthesis functional group at day 12 of adipogenesis. Values are expressed as mean of 16 differentially expressed genes in the triacylglycerol synthesis functional group as indicated by Ingenuity Pathway Analysis ± SEM of 3 age and BMI pair-matched normal-weight PCOS and control women

**Table 1:** Total content and de novo synthesis of FAs and cholesterol

	FA type	Group (*p* values)	Time (*p* values)	Group * time (*p* values)
Total production	Total C14:0	**0.019**	**0.012**	0.066
	Total C16:0	**0.019**	**0.013**	0.068
	Total C16:1	**0.028**	**0.016**	0.089
	Total C18:1	**0.033**	**0.024**	0.159
	Total C18:2	**0.050**	**0.038**	0.273
	Cholesterol	0.580	**0.045**	0.339
De novo synthesis	De novo synthesis C14:0	**0.009**	**0.005**	0.094
	De novo synthesis C16:0	**0.012**	**0.007**	0.071
	De novo synthesis C16:1	**0.026**	**0.019**	0.196
	De novo synthesis C18:1	**0.020**	**0.012**	0.152
	Cholesterol	0.067	**0.035**	0.254

Group represents PCOS versus control difference. Time represents day 7 versus day 12 difference. Group * time represents the interaction term between female group and time (day 7 vs day 12). Bold values indicate *p* < 0.05

Transcription factor binding motifs (TFBM) were analyzed to identify putative TFs whose binding may have been influenced by changes in chromatin accessibility. TFBMs that were significantly enriched at the differentially accessible regions (*q* < 0.05) at days 0, 3, and 12 highly involved the activator protein-1 (AP-1) sub-family among the top 20 TFBMs, including Fra1, Atf3, BATF, Fra2, JunB, AP-1, and Fosl2, all of which are implicated in adipogenesis [24, 26]. All significantly enriched TFBMs at each time point and their motif sequence are reported in Additional file 3: Supplemental Table 2.

Of interest, TFBM enrichment belonging to PPAR and CEBP proteins in differential ATAC-seq peaks was identified. The PPAR and CEBP families of TFs include master regulators of adipogenesis, PPAR*γ*, and CEBP*α*, suggesting that binding of these TFs may be affected by PCOS-related changes in chromatin accessibility (Additional file 3: Supplemental Table 1). Furthermore, we found regions of differential chromatin accessibility (PCOS vs control) overlapping the genes that encode for the PPAR*γ* and CEBP*α* TFs at day 0 and 12, respectively (Additional file 2: Supplemental Fig. 5). This observation indicates that PCOS-related chromatin accessibility changes may influence regulation of expression of master adipogenesis regulators, such as *PPARγ* and *CEBPα*, at specific time points.

**Fig. 5:**
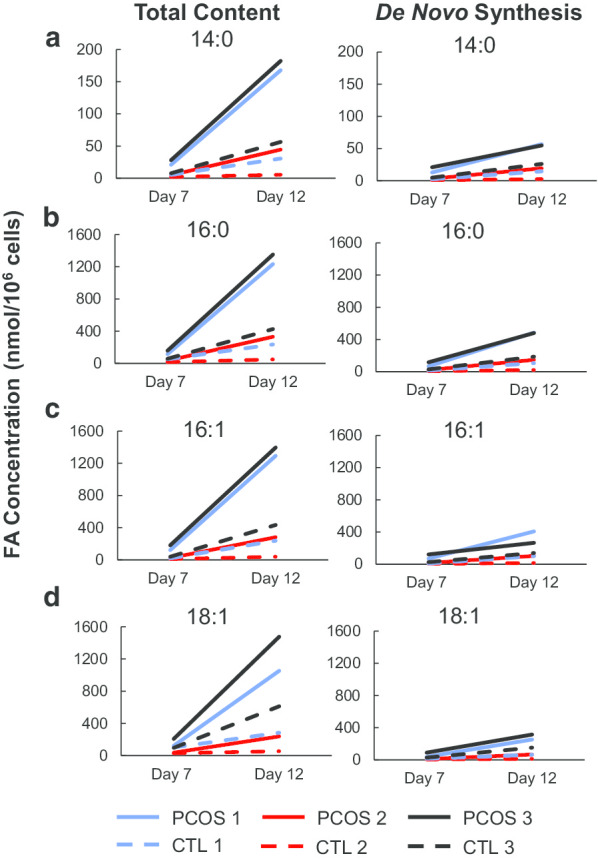
Total content and de novo FA synthesis at days 7 and 12 of adipogenesis. Total content and de novo synthesized FA in newly-formed adipocytes of each pair-matched sample for **a** C14:0 myristic acid, **b** C16:0 palmitic acid, **c** C16:1 palmitoleic acid, and **d** C18:1 oleic acid. Values are expressed as nanomoles of FA per one million cells. Each line represents one study patient. Women with PCOS are represented as solid lines, and control women as dotted lines. Each color represents each age- and BMI- pair matched sample

While no enrichment in gene ontology (GO) terms associated with differentially accessible regions was identified for days 0 and 12, differential peaks at day 3 were significantly enriched in a few pathways based on GO cellular component, including MHC protein complex, integral component on luminal side of endoplasmic reticulum membrane, MHC class I protein complex, ER to Golgi transport vesicle membrane, ER to Golgi transport vesicle, endocytic vesicle membrane, and coated vesicle membrane (Additional file 3: Supplemental Table 3), suggesting a possible relationship between cell structural/morphological changes and chromatin accessibility changes during adipogenesis.

### RNA expression levels and related gene functions and pathways

RNA-seq analyses were performed to determine whether gene expression changes accompany the shift in chromatin accessibility observed in PCOS compared to control samples. A total of 4157 genes were differentially expressed (1706 genes upregulated; 2451 genes downregulated) with at least a twofold change throughout days 0, 3, 7, and 12 of SC abdominal ASCs of PCOS versus control women undergoing adipogenesis. Specifically, 31, 466, 768, and 441 genes were upregulated in PCOS on days 0, 3, 7, and 12, respectively, while 130, 742, 974, and 605 genes were downregulated on days 0, 3, 7, and 12, respectively. Unsupervised principal component analysis of RNA-seq data (Additional file 2: Supplemental Fig. 2B) assessed the separation between female groups and time points, and indicated global gene expression profiles and sample distributions that separated both female groups at each time-point when using only a one-dimensional level (PC1 = 44%). Female types differences were distinctly separated at day 0 and day 3, while at day 7 and day 12 this separation was less clear.

Using activation *z*-score > 2 in combination with *p* < 0.05 for more stringent analysis, Ingenuity Pathway Analysis (IPA) identified *TGFβ1*, an adipogenic inhibitor, as a putative upstream regulator of differentially expressed genes consistently across all late-stage adipogenesis time points at days 3, 7, and 12, and showed decreased *TGFβ1* expression in PCOS cells compared to control cells (*p* < 7.6 × 10^–9^, *z* > 2.0, days 3, 7, 12), consistent with enhanced adipogenesis previously observed in PCOS ASC-derived adipocytes [25]. At day 12 of adipogenesis, *TGFβ1* may be involved in the regulation of several differentially expressed genes, including *PPARγ*, *CEBPα*, *JUNB*, and *FOS* among others (see Additional file 2: Supplemental Fig. 6).

**Fig. 6:**
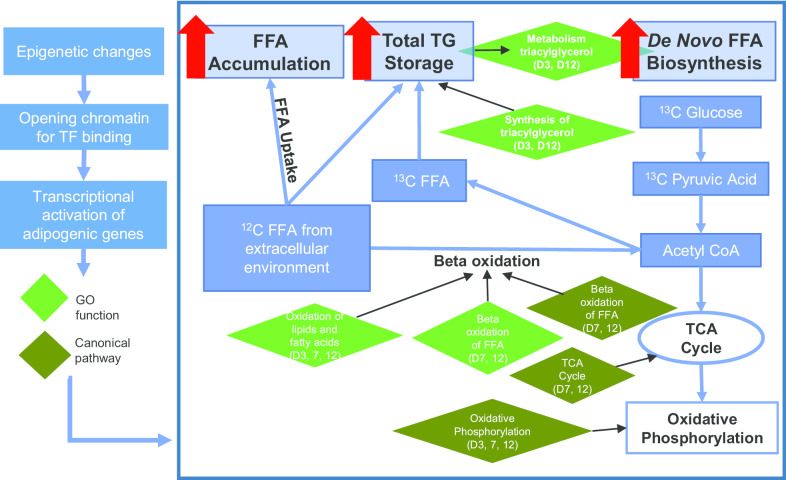
Schematic model of fatty acid composition experiments and potential effects from epigenetic changes that may influence chromatin accessibility, gene transcription, and energy metabolism processes in newly-formed adipocytes from normal-weight PCOS women. Diamond shapes represent GO functions (green) and canonical pathways (dark green) identified as significantly upregulated in PCOS by Ingenuity Pathway Analysis

While gene ontology analysis using IPA software did not identify functional groups or canonical pathways associated with the small set of differentially expressed genes at day 0, the top ten functional groups identified at days 3, 7, and 12 related to lipid and FA metabolism ranked by activation *z*-score of differentially expressed genes at each time point were: fatty acid oxidation, lipid oxidation, triacylglycerol synthesis, and triacylglycerol metabolism (day 3, *p* < 1.3 × 10^–6^, *z* > 2 for all values; Fig. 3a); lipid oxidation, fatty acid oxidation, acylglycerol synthesis, and fatty acid beta-oxidation (day 7, *p* < 5.5 × 10^–5^, *z* > 2 for all values; Fig. 3b); and lipid oxidation, fatty acid oxidation, acylglycerol synthesis, acylglycerol metabolism, triacylglycerol synthesis, triacylglycerol metabolism, and fatty acid beta-oxidation (day 12, *p* < 1.6 × 10^–4^, *z* > 2 for all values; Fig. 3c).

Similarly, among the ten most biologically relevant and significant canonical pathways related to energy metabolism at days 3, 7, and 12 were: oxidative phosphorylation, triacylglycerol degradation, super-pathway of cholesterol biosynthesis, and triacylglycerol biosynthesis (day 3, *p* < 3.0 × 10^–2^, *z* > 2.0 for all values; Fig. 3d); oxidative phosphorylation, TCA cycle II, fatty acid beta-oxidation I, and super-pathway of cholesterol biosynthesis (day 7, *p* < 3.2 × 10^–2^, *z* > 2.0 for all values; Fig. 3e); and oxidative phosphorylation, super-pathway of cholesterol biosynthesis, fatty acid beta-oxidation I, triacylglycerol degradation, ketogenesis, and TCA cycle II (day 12, *p* < 3.9 × 10^–2^, *z* > 2.0 for all values; Fig. 3f). These upregulated GO functional groups and canonical pathways in PCOS were further confirmed and identified by GSEA, with enriched cellular processes including oxidative phosphorylation, adipogenesis, and fatty acid metabolism (NES > 6.1, *p* < 0.001; all processes).

### Chromatin accessibility and RNA expression of critical genes related to adipocyte function and triacylglycerol synthesis

Significantly upregulated critical genes governing adipocyte cell differentiation and function in newly-formed adipocytes from SC abdominal ASCs of PCOS versus control women were identified at days 3, 7, and 12, including *PPARγ*, *CEBPα*, *AGPAT2*, *ADIPOQ*, *PLIN1*, *SLC2A4* (Fig. 2, right panel), *FABP4* and *LPL* (Additional file 2: Supplemental Fig. 7A–B, right panel). Interestingly, the chromatin accessibility of each of these eight respective genes generally peaked at day 3 and decreased by day 12 of adipogenesis (Fig. 2 and Additional file 2: Supplemental Fig. 7A–B, left panels), while RNA expression increased until day 7 or day 12 (Fig. 2 and Additional file 2: Supplemental Fig. 7A–B, right panels). Conversely, a transcriptional repressor of the androgen receptor (AR), *ANKRD1*, as well as genes involving the Wnt signaling pathway, *FZD1*, *SFRP1*, and *WNT10B*, were downregulated in PCOS cells compared to control cells (Additional file 2: Supplemental Fig. 7C–F, right panel). The chromatin accessibility of *ANKRD1*, *FZD1*, *SFRP1*, and *WNT10B* were comparable between PCOS and control samples and generally increased at day 3 followed by decreased accessibility by day 12 (Additional file 2: Supplemental Fig. 7C–F, left panels). The increase in chromatin accessibility followed by increased RNA expression at the next time point may indicate that opening of the chromatin and binding of transcription factors precede transcription.

In order to evaluate the relationship between differential chromatin accessibility, transcriptome, and potential fatty acid synthesis function in newly-formed adipocytes, we performed a combination analysis with both differential chromatin accessibility and expression of genes found to be part of the triacylglycerol synthesis GO functional group at day 12 of adipogenesis (*AGPAT2*, *BSCL2*, *DGAT1*, *DGAT2*, *FASN*, *FITM2*, *GPAM*, *GPAT3*, *IL6*, *LPIN1*, *LPL*, *MOGAT1*, *NRIH3*, *PNPLA2*, *PNPLA3)* (see Additional file 4). The increase in differential chromatin accessibility was comparable to the increase in RNA expression in this set of genes (values are expressed as mean of 15 differentially expressed genes in the triacylglycerol synthesis functional group) (Fig. 4), suggesting a relationship between chromatin accessibility, transcriptome, and fatty acid synthesis function as part of an epigenetic regulated mechanism governing adipogenesis in PCOS women. In addition, when mean chromatin accessibility and RNA expression of these 15 differentially expressed genes were assessed at earlier time points, mean chromatin accessibility at these regions peaked at day 3, while RNA expression increased beginning at day 0 to maximally at day 7 (data not shown). When these 15 genes implicated in the triacylglycerol synthesis functional group were analyzed individually, there was an increase in differential chromatin accessibility at day 3 followed by increased RNA expression at day 7 in a majority of these genes (Additional file 2: Supplemental Fig. 8), consistent with previously observed differential chromatin accessibility and TF binding patterns in genes governing adipogenesis and adipocyte function (Fig. 2, Additional file 2: Supplemental Fig. 7),

### Total content and de novo synthesis of FAs in newly-formed adipocytes

Based on increased expression of genes related to fatty acids (FAs), lipid, and energy metabolism processes found in newly-formed adipocytes of PCOS ASCs, total content, and de novo synthesis of FAs at days 7 and 12 of adipogenesis, the earliest time points in which lipid droplets were detected based on our previous study [25], were subsequently determined in these cells via isotopic spectral analysis (ISA) of newly-formed adipocytes that were cultured in labeled ^13^C-glucose adipogenic differentiation medium 48 h prior to harvest. The four most abundantly produced FAs (total content and de novo synthesis) by the adipocytes at days 7 and 12 of adipogenesis were myristic acid (C14:0), palmitic acid (C16:0), palmitoleic acid (C16:1), and oleic acid (C18:1) of the six FAs analyzed (data not shown). There was a significant difference between day 7 and day 12 in the total content, as well as de novo synthesis of these four FAs in both PCOS and control adipocytes (Fig. 5). Significant differences were also detected between female groups (PCOS vs control) as well as between day 7 and 12 (time effect) for newly-formed adipocytes in total and de novo synthesis for C14:0, C16:0, C16:1, and C18:1 (*p* < 0.05, all four; Table 1, Fig. 5). Total content of C18:2 tended to differ between PCOS and control cells (*p* = 0.05, Table 1), while demonstrated significant differences between day 7 and 12 (*p* < 0.05, Table 1). Significant differences also were observed between day 7 and 12 in total and de novo synthesis of cholesterol (*p* < 0.05, Table 1). No significant interaction term between female group (PCOS vs control) and time (day 7 vs day 12) was observed in any of the analyzed FAs.

At day 12 of adipogenesis, PCOS adipocytes had significantly increased total content and de novo synthesis of C14:0, C16:0, C16:1 and C18:1 compared to age and BMI-matched control cells (*p* < 0.03 for all four FAs, Fig. 5). Furthermore, at day 12, total content and de novo synthesis in newly-formed adipocytes derived from SC abdominal ASCs of PCOS subjects were at least twofold greater than those from each age- and BMI-matched control subject (Additional file 2: Supplemental Fig. 9). There was also a significant increase in de novo synthesis of cholesterol at day 12 (*p* < 0.05), without significant differences in total C18:2 (data not shown). No significant differences between each pair-matched PCOS and control cells were observed at day 7. These significant increases in FA content and synthesis from day 7 to day 12 of adipogenesis as well as in PCOS relative to control cells at day 12, demonstrate functional differences in newly-formed adipocytes of PCOS versus controls that accompany increased chromatin accessibility and RNA expression found in genes related to the triacylglycerol synthesis functional group.

## Discussion

The present paper shows a distinct pattern of chromatin remodeling in SC abdominal ASCs of normal-weight PCOS women versus age-and BMI-matched controls during adipogenesis in vitro. Specifically, in PCOS compared to control ASCs, limited chromatin accessibility at day 0 (quiescent stage) was followed by exaggerated availability (active stage) after exposure to adipogenic media for 12 days without androgen exposure (Fig. 1). These findings suggest a unique pattern of chromatin remodeling in PCOS ASCs during adipogenesis that could modify transcription of early- and late-stage adipogenic genes related to adipocyte function, especially if related to *PPARγ* and *CEBPα* binding regions [28–30]. Interestingly, changes in chromatin accessibility over time in all stem cells mostly involved promoter regions (Fig. 1), suggesting dynamic rewiring of promoter-anchored chromatin loops to reprogram enhancer activity and change target gene expression perhaps in response to extracellular conditions (i.e., exposure to adipogenic media) like that seen in 3T3-L1 cells [31] to promote adipocyte function.

In SC abdominal ASCs of normal-weight PCOS women compared to controls, the significant enrichment over time of motifs from the AP-1 family (c-Jun, c-Fos, Jun-B, Fos-B, and Fra-1), a critical TF involving adipocyte differentiation [32, 33] implies their critical roles in accelerated adipogenesis in vitro, as previously described in PCOS ASCs [25] (Additional file 3: Supplemental Table 2). These binding regions remained more accessible from day 3 to late-stage adipogenesis (day 12) in PCOS ASCs relative to those of controls, although similar binding regions in 3T3-L1 cells are accessible during early-stage more so than late-stage adipogenesis [34], perhaps due to species differences in cell signaling, since human adipocytes secrete paracrine factors (i.e., hormones, cytokines) that facilitate ASC commitment to preadipocytes and their differentiation into mature adipocytes [35, 36]. In support of this, co-culture of adipocytes with ASCs and macrophages from the stromal vascular fraction (SVF) of human adipose promotes adipocyte differentiation [37], suggesting paracrine signaling through cell–cell contact and/or secreted products that may favor pro-adipogenic TF activity through AP-1. In PCOS, *FOS*, as a heterodimer of the AP-1 TF, is critical in metabolic and reproductive function [38], therefore strengthening the role of FOS/AP-1 in mediating adipocyte signaling for accelerated adipogenesis.

Despite a different temporal pattern of chromatin accessibility in PCOS ASCs relative to controls, a global increase in accessibility of gene promoters at day 3 in all cells corresponded with morphological and functional changes as preadipocytes differentiated into newly-formed adipocytes [13]. Interestingly, differences in PCOS versus control chromatin accessibility at this time were significantly enriched in secretory pathways facilitating formation of exosomes (Additional file 3: Supplemental Table 3) as nanovesicles that facilitate cell–cell communication through soluble factors and microRNAs [39]. Therefore, exosomes, as known regulators of mesenchymal stem cell differentiation [40, 41], could act in concert with chromatin accessibility to differentially alter the function of PCOS ASCs during adipogenesis [35–37].

Surprisingly, the patterns of DNA methylation were similar between PCOS and control ASCs (data not shown), as opposed to differential DNA methylation patterns previously noted in PCOS-like sheep models, monkey models and in human PCOS adipose and granulosa cells [22, 23, 42, 43]. However, these discrepancies between our data and previous study findings may be related to differences in species, tissue type, fat depot, human adipose tissue versus a homogenous population of ASCs, and/or the BMI of each respective study. Furthermore, our PCOS cohort is within a healthy, normal weight range to eliminate the confounding effects of age and obesity on insulin resistance.

Prenatally T-treated monkeys and sheep have been essential animal models used to program a permanent PCOS–like phenotype since tissue differentiation in these species is completed during fetal life (i.e., precocial species), as in humans [9]. Interestingly, while we did not detect changes in AR gene expression in the present cells, our transcriptome data identified *ANKRD1*, a transcriptional repressor of the androgen receptor (AR), to be significantly downregulated in newly-formed PCOS adipocytes at days 7 and 12 (Additional file 2: Supplemental Fig. 7C, right panel). Furthermore, *ANKRD1* can repress AP-1 binding sites [44], agreeing with enhanced AP-1 binding motif accessibility in PCOS ASCs as early as day 3. These changes in chromatin accessibility during adipogenesis in ASCs from PCOS women in vitro did not require the presence of androgen in the culture medium, which suggests intrinsic changes in PCOS ASCs in combination with a hyperandrogenic milieu as critical integrated players during developmental programming of PCOS.

Altered chromatin accessibility in PCOS ASCs may also have contributed to the enhanced expression of adipogenic/metabolic genes controlling lipid storage and oxidation between day 3 and day 12 of adipogenesis in vitro. In fact, differential peaks overlapping key regulators of adipogenesis, *PPARγ* and *C/EBPα* [24], were identified at day 0 and day 12, respectively (Additional file 2: Supplemental Fig. 5), in addition to up-regulation of RNA levels at days 3–12 found in PCOS cells (Fig. 2a, b), indicating regulatory changes in *PPARγ* and *C/EBPα* genes to be reflected in both chromatin accessibility and transcript abundance. *AGPAT2*, which induces the expression of *PPARγ* and *C/EBPα* [45], as well as critical genes governing adipocyte function such as *ADIPOQ*, *PLIN1*, *SLC2A4*, *FABP4* and *LPL* were also up-regulated in PCOS cells with peak expression level at days 7–12 following peak chromatin accessibility generally at day 3 (Fig. 2, Additional file: Supplemental Fig. 7), suggesting chromatin opening and TF binding to precede and potentially influence transcription at these genes. Simultaneously, several genes of the Wnt signaling pathway, specifically *FZD1*, *SFRP1*, and *WNT10B*, were consistently downregulated in PCOS cells over time (Additional file: Supplemental Fig. 7D–F, right panel), with the role of Wnt10B in stabilizing *β*-catenin to inhibit glycogen synthase kinase 3 and adipogenesis [46] and in suppressing Wnt receptors, including Fzd1, Fzd2, and Fzd5, during adipocyte differentiation [47].

*TGFβ1* signaling normally inhibits adipocyte differentiation through repression of *C/EBPβ* and *C/EBPδ* [48, 49]. In our study, downregulation of *TGFβ1* signaling was identified as the primary upstream regulator of differentially expressed genes at days 3, 7, and 12 (Additional file 2: Supplemental Fig. 6), agreeing with enhanced adipogenesis and exaggerated lipid accumulation in newly-formed adipocytes of normal-weight PCOS women in vitro [25]. In PCOS, *TGFβ* dysregulation contributes to several endocrine-metabolic changes, while reproductive dysfunction is a feature of *TGFβ*-knockout mice [50]. Therefore, downregulation of *TGFβ1* signaling may promote adipogenesis via altered adipogenic gene expression to modify the PCOS phenotype [51].

The top GO functions and pathways associated with differentially expressed genes between PCOS and control newly-formed adipocytes included fatty acid and lipid oxidation, as well as oxidative phosphorylation and the TCA cycle (Fig. 3), indicating more efficient energy utilization processes alongside accelerated differentiation in these cells. In agreement, our functional studies found increased total content and de novo synthesis of the four most abundant FAs (i.e., myristic, palmitic, palmitoleic, and oleic acid) at days 7 and 12 in newly-formed PCOS adipocytes compared to controls (Fig. 5). Importantly, saturated FAs, such as palmitic and myristic acid, are implicated in lipotoxicity through endoplasmic reticulum stress and apoptosis [52], while unsaturated FA de novo synthesis, including oleic and palmitoleic acid, protects against lipotoxicity through triglyceride synthesis and storage [53, 54]. Therefore, the increased overall total content combined with equal de novo synthesis of saturated and unsaturated FAs in newly-formed PCOS versus control adipocytes points to accelerated lipid accumulation in PCOS cells within the time frame of our in vitro studies.

These enhanced energy-related processes in newly-formed adipocytes of SC abdominal ASCs from normal-weight PCOS women may be modulated by increased chromatin accessibility and transcription of genes, as seen in the parallel increase at day 12 between chromatin accessibility and transcription of genes implicated in the triacylglycerol synthesis functional group (Fig. 4). Additionally, in a majority of this set of genes, increased chromatin accessibility generally preceded elevated RNA expression (Additional file 2: Supplemental Fig. 8) similar to that of genes implicated in adipogenesis and adipocyte function (Fig. 2, Additional file 2: Supplemental Fig. 7). Triacylglycerol synthesis and FA synthesis are intricately related through the common triglyceride storage (Fig. 6). Therefore, differential chromatin and gene regulation patterns of the triacylglycerol synthesis functional group could also influence the increased FA synthesis observed in PCOS newly-formed adipocytes. Ultimately, these chromatin remodeling patterns during adipogenesis with enhanced adipogenic gene expression and lipid accumulation (total FA content and synthesis) suggest a cellular program that promotes more efficient fat storage, as illustrated in Fig. 6. These metabolic changes in PCOS stem cells in vitro in combination with a hyperandrogenic environment in vivo may represent an ancestral trait that originally favored fat storage, when maternal food deprivation programmed enhanced adipogenesis in the fetus to meet metabolic and reproductive demands in later life through optimal fat storage and energy utilization [9, 55, 56]. If so, these same PCOS-related traits of efficient fat storage operative within today’s obesogenic environment could lead to increased risks for metabolic dysfunction and subfertility in some PCOS women through lipotoxicity [57].

## Conclusions

SC abdominal ASCs from normal-weight PCOS women undergoing adipogenesis in vitro demonstrate dynamic changes in chromatin accessibility related to adipocyte differentiation that may be part of cellular programming. These chromatin remodeling patterns accompany increased activation of genes related to adipogenesis and adipocyte function, specifically lipid oxidation, TCA cycle, and oxidative phosphorylation, as well as increased total content and de novo synthesis of both saturated and unsaturated FAs in PCOS cells, suggesting coordinated correspondence between chromatin accessibility, transcriptome, and fatty acid synthesis function as part of an epigenetic-regulated mechanism governing adipogenesis in PCOS women. Our data agree with previous prenatally T-treated animal models of PCOS in precocial species and adult female monkeys with natural hyperandrogenemia and PCOS-like traits [8, 58, 59]. Our findings also expand on developmental programming of PCOS stem cells as a potentially favorable, evolutionary mechanism to protect against food deprivation during low caloric intake that in today’s environment of excess caloric intake versus energy utilization increases the risk of developing metabolic disease. While it may be premature to draw definitive clinical conclusions about the complex relationships of PCOS stem cell function in vitro with the endocrine-metabolic environment of PCOS in vivo, our pilot study represents the first to our knowledge in healthy normal-weight PCOS women and age- and BMI-matched controls to integrate chromatin accessibility, gene expression, and FA content and synthesis in SC abdominal ASCs during adipogenesis in vitro. In doing so, it has identified PCOS-related specific TFs and their binding sites as well as downstream target genes controlling FA content and synthesis that may open new avenues for future mechanistic studies on the developmental programming in PCOS as well other metabolic-related diseases.

## Methods

### Study participants

Approval by the UCLA Institutional Review Board was obtained. Our cohort of eight NIH-defined PCOS women and age/BMI-matched normoandrogenic ovulatory women (controls) who had participated in our NIH study of adipose stem cell characteristics in PCOS [25] could be clearly separated into two groups, based on molecular and functional outcomes specifically in lipid accumulation and *PPARγ* and *CEBPα* gene expression. From this cohort, three normal-weight PCOS and three age- and BMI-matched control women (all women, 19–33 years; 19.5–23.9 kg/m^2^) were selected based on significant divergence in lipid accumulation and *PPARγ* and *CEBPα* overexpression [25], as defined by at least a 2.0-fold increase of *PPARγ* and *CEBPα* gene expression in newly-formed adipocytes (day 12) compared to age- and BMI-matched controls (Additional file 1: Supplemental Fig. 1). Selection and exclusion criteria of all subjects and blood sampling as well as hormonal assays were as previously described [25].

### SC abdominal fat biopsy and isolation of ASCs

Each subject (3 PCOS; 3 controls) underwent a SC fat biopsy whereby approximately 0.5–1 g of freshly isolated SC adipose was obtained through a 1 cm lower abdominal incision. SC adipose was washed with DMEM and digested at 37 °C in DMEM (Corning, Tewksbury, MA) containing 0.075% collagenase (Sigma-Aldrich, St Louis, MO) for 45–60 min on a shaker to release all cells present in the adipose tissue including adipocytes and the stromal-vascular fraction (SVF). Digested material was then filtered through a 150-μm mesh, washed with DMEM/10% fetal calf serum (FCS) (Corning, Tewksbury, MA), and centrifuged (200×*g*, 2 min) to separate adipocytes present in the top layer of the supernatant. Subsequent centrifugation (800×*g*, 10 min) led to obtaining a cell pellet containing the SVF which was then washed in DMEM/10% FCS and plated in 60 mm dishes containing DMEM/10% FCS, 0.05 U/ml penicillin, 0.05 mg/ml streptomycin, 1.25 mg/ml fungizone and cultured at 37 °C until confluency in 5% CO_2_. First- and second-generation ASCs from each patient were frozen and kept in liquid nitrogen for use in subsequent ASC studies.

### Cell culture

Second-generation ASCs were expanded to fourth generation in DMEM/10% FCS, 0.05 U/ml penicillin, 0.05 mg/ml streptomycin, and 1.25 mg/ml fungizone and incubated in adipocyte differentiation medium (DMEM/Ham’s F-12 (1:1, v/v), HEPES pH 7.4, fetal bovine serum, biotin, pantothenate, human insulin, dexamethasone, 3-Isobutyl-1-methylxanthine (IBMX), PPARγ agonist, penicillin, streptomycin, amphotericin B [Zen-Bio, Research Triangle Park, NC]) without androgen exposure for 12 days to induce ASC differentiation into adipocytes. Culture medium was changed every 48 h. At days 0, 3, and 12, cells were trypsinized and 100,000 cells were subject to nuclei preparation for Assay for Transposase-Accessible Chromatin-sequencing (ATAC-seq). At days 0, 3, 7, and 12, RNA lysate was extracted from one 60-mm dish for RNA-sequencing (RNA-seq).

For fatty acid analysis, ASCs were expanded to fifth generation prior to induction of adipogenesis via 12-day incubation in 12-well plates containing adipocyte differentiation medium (ZenBio, Inc, Research Triangle Park, NC). All samples were cultured in quadruplicates (four wells per sample) for more robust analysis. 48 h prior to harvesting newly-formed adipocytes at days 7 and 12 of adipogenesis, the earliest time points in which lipid droplets were detected based on our previous study [25], cells were incubated in custom adipocyte differentiation medium containing 3.15 g/L solution of labeled ^13^C-glucose (Cambridge Isotope Laboratories, Tewksbury, MA) to quantify de novo synthesis of fatty acids. At days 7 and 12, harvested newly-formed adipocytes were lysed in 75 μL of 6 M aqueous guanidine-HCl for 3 to 5 min. All quadruplicate samples were then pooled into one well and 150 μL of 3 M methanolic guanidine was added to each pooled well and 50 μL of this mixture was transferred into a glass tube and frozen at − 80 °C until fatty acid compositional analysis. In parallel, a set of adipocytes cultured simultaneously under identical conditions were lifted with trypsin and counted using a Cellometer Vision CBA (Nexcelom Bioscience Inc, San Diego, CA) to estimate the number of cells harvested for fatty acid compositional analysis.

### ATAC-Seq library preparation

ATAC-seq libraries were generated according to Buenrostro et al. [60] with minor modifications. Nuclei were treated with Tn5 transposon TDE1 (Nextera DNA Library Prep kit, Illumina, San Diego, CA) for 35 min at 37 °C. The transposition reaction was then purified using Zymo DCC-5 columns (Zymo Research, Irvine, CA). Following, Polymerase Chain Reaction (PCR) was performed using 25 μl NEBNext High-Fidelity 2 × PCR Master Mix (NEB, MA, Boston) 4 µl of each Index 1 (i7) and index 2 (i5) (Nextera XT Index kit, Illumina, San Diego, CA), 2 µl of PPC (Illumina, San Diego, CA) and 15 µl of purified tagmentation reaction. After performing the initial extension and 5 PCR cycles, quantitative PCR was performed on 5 µl of the previous PCR reaction by adding 1 µl of SYBR Green (15 ×), 1 µl of PPC, 5 μl NEBNext High-Fidelity 2 × PCR Master Mix and 3 µl of H2O in a final volume of 15 µl for 20 cycles. The number of additional cycles was determined as the cycle number corresponding to ¼ of maximum fluorescent intensity. Final libraries were purified using 45 µl (1:1 ratio) of SPRI beads (Beckman and Coulter, Indianapolis, Indiana) cat# B23318). Final libraries were visualized on a TapeStation 2200 using a D1000 ScreenTape. Libraries were pooled and paired-end sequenced (PE 2X100) on the Illumina HiSeq4000.

### Quality control of ATAC-Seq libraries

Quality assessment of the raw sequencing reads was performed using FastQC (v0.11.8) [61]. Adapter sequences and low-quality sequences were trimmed using Trimmomatic (v0.39) [62]. Reads that were shorter than 36 base pairs (bp) after trimming were removed. The remaining trimmed reads were further trimmed to a maximum read length of 50 bp to rescue the apparent drop in 100 bp fragments observed after the first alignment attempt. These trimmed 50 bp reads were then aligned to the human hg19 (hg37) reference genome from Ensembl with Bowtie2 (v2.3.5) [63]. The "−x 2000" parameter was specified to allow the potential alignment of fragment lengths up to 2000 bp (recommended with ATAC-seq). Reads were then filtered to remove reads mapping to the mitochondrial genome, reads with a mapping quality score < 30, improperly paired reads as defined by samtools 0 × 02 flag, and duplicate reads as determined by Picard Tools [64].

Following alignment and read filtering, deeptools (v 3.3.1) [65] was used to investigate genome-wide ATAC-seq read coverage patterns and assess overall Pearson correlation across libraries. The ATAC-seq signal, measured in reads per genome coverage (RGPC), was visualized in 6-Kb windows centered at all gene transcription start sites (TSS) based on the Ensembl gene annotation (GRCh37.75). Summary statistics for all ATAC-seq libraries are reported in Additional file 3: Supplemental Table 4.

### ATAC-Seq chromatin accessibility analysis

Regions of accessible DNA (ATAC-seq peaks) were identified for each sample using MACS2 (v2.1.2) [66] with a significance threshold *p* value < 0.05. Differential analysis of peak regions was performed using DiffBind (v2.12.0) [67] with the DESeq2 framework. Briefly, a consensus peak set was generated first, by taking the union of peaks found in at least two samples. Following, peaks 150 bp on either side of their center were extended so that all of the consensus peaks were 300 bp long. Peaks located on unplaced hg19 assembly contigs were removed, resulting in a final set of 1,078,442 consensus ATAC-seq peaks, which were used for downstream comparisons.

Three pairwise comparisons were performed to find significant (FDR *p* value < 0.05) regions of differential accessibility between PCOS and control samples at days 0, 3, and 12 (each unique combination of condition/time point had three replicates) while pair match was used as a blocking factor in the model. DiffBind was used to perform principal component analysis (PCA) of samples based on the read counts in the consensus peak regions. Lastly, ChIPseeker (v 1.22.0) [68] with default parameters assigned differential peaks to nearby genes based on Ensembl hg19 gene annotation (GRCh37.75).

To identify putative TFs whose binding may have been influenced by changes in chromatin accessibility, HOMER (v4.11.1) with the “-size given” parameter was used to identify transcription factor-binding motifs enriched among differential peaks from each of the three pairwise comparisons. Lastly, to identify candidate gene ontology terms affected by differential chromatin accessibility, pathway analyses of differential peaks were performed using the Genomic Regions Enrichment of Annotations Tool (GREAT) [69].

### RNA library preparation and sequencing

Total cellular RNA was isolated using RNeasy mini kit (Qiagen, Carlsbad, CA) according to the manufacturer’s protocol. RNA quality was checked at the TapeStation using an RNA ScreenTape. 500 ng of total RNA were processed using the KAPA HyperPrep mRNA kit (Roche, Pleasanton, CA) according to the manufacturer's instructions. Briefly, mRNAs were captured with oligo-dT magnetic beads and fragmented for 6 min at 94 °C. First strand synthesis was performed on fragmented RNA, followed by second strand synthesis and dA-tailing. Adapter ligation was performed using KAPA single-indexed adapters (KAPA, Pleasanton, CA). The adapter-ligated fragments were amplified with 10 PCR cycles. Final libraries were then pooled and sequenced on a HiSeq4000 single-end 50 nt.

### RNA-Seq data analysis

Sequencing reads were first mapped to the latest UCSC transcriptome set using Bowtie2 version 2.1.0 [63], and the gene expression level was estimated using RSEM v1.2.15 [70]. TMM (trimmed mean of *M* values) was used to normalize gene expression. Differentially expressed genes with greater than twofold change and *p* < 0.05 (unpaired *t* test) were identified using the EdgeR program [71]. Pair match samples were taken into account by performing paired test during differential analysis. The most significant functional groups, canonical pathways, and networks were identified using Qiagen’s Ingenuity Pathway Analysis (IPA). In addition to *p* < 0.05, *z*-score > 2 (indicating ≥ 99% confidence) was also used in pathway analyses to further emphasize the likelihood of differentially expressed genes belonging in specific functional groups, canonical pathways, and networks. Fischer's exact test with false discovery rate (FDR) option was also used to calculate the significance of canonical pathways. A complete dataset from RNA-seq analyses is included in Additional file 4.

### Gene set enrichment analysis

Differential gene expression testing was performed by DESeq2 (v1.10.1) [72], and resulting fold change values were used as a pre-ranked gene list for gene set enrichment analysis (GSEA 4.0.1) [73]. ‘Classic’ mode was used for enrichment statistic calculation. For unbiased discovery of enriched gene sets, the HALLMARK gene sets were used as queries [74].

### Fatty acid compositional analysis

Total content and de novo FAs (myristic acid, C14:0; palmitic acid, C16:0; palmitoleic acid, C16:1; stearic acid, C18:0; oleic acid, C18:1; and linoleic acid, C18:2) and cholesterol were measured as previously described [75]. Briefly, an acid methanolysis reaction was carried out to convert free and esterified fatty acids into methyl esters. The resulting methyl esters and cholesterol were extracted in hexane and analyzed by gas chromatography-mass spectrometry (GC–MS). Lipids were normalized against internal standards and quantified against an external standard curve. Isotope distributions were measured in SIM mode and labeling patterns were analyzed using Isotopic Spectral Analysis (ISA). Total content of FA was defined as the sum of accumulation and de novo synthesis of synthesized FAs from acetyl-CoA originating from various carbon sources (natural as well as from ^13^C-glucose). De novo synthesis of FAs was calculated by multiplying the percentage of synthesis (ISA) by the total amount of each FA (nmol)/10^6^ cells.

Linear mixed effects models for each FA were run with terms for group (PCOS vs control), time (day 7 and 12), and the group * time interaction with a random effect for pair match [1–3]. From these models, an overall group effect, time effect, or a differential time effect by group interaction can be estimated. Pairwise contrasts were extracted from the model to estimate differences between pair match samples at each time point. Models were run using SAS V9.4 (Cary, NC) and *p* values < 0.05 were considered statistically significant.

## Supplementary information

**Additional file 1:** Additional figure of *PPARγ* and *CEBPα* expression of 8 PCOS versus 8 age- and BMI-matched control women from our previous study (Fisch, 2018). Supplemental Fig. 1. Fold changes of *PPARγ* and *CEBPα* gene expression levels at day 12 of adipogenesis. Values represent PCOS versus control. Three out of eight total pair match subjects were selected for the present study based on significantly higher (> 2.0-fold) gene expression levels of *PPARγ* and *CEBPα* in PCOS.

**Additional file 2:** Additional figures with data from ATAC-seq and RNA-seq. Supplemental Fig. 2. PCA plots from 3 PCOS and 3 control samples of (A) ATAC-seq data at days 0, 3, and 12 of adipogenesis and (B) RNA-seq data at days 0, 3, 7, and 12 of adipogenesis. Time points are labeled using different colors while female-type is labeled using different shapes. Supplemental Fig. 3. Heatmaps of global chromatin accessibility changes at gene transcription start sites (TSS) in PCOS and control samples across time. ATAC-seq read density in each sample within a 6 kb window centered at all annotated TSS in the genome are shown from high (blue) to low (red) read densities. Higher ATAC-seq read density at TSS (e.g. day 3) suggests higher chromatin accessibility. Above each heatmap, a profile plots summarizes the normalized Reads Per Genome Content (RPGC) across all TSS. Supplemental Fig. 4. Venn diagram of the number of significant differentially accessible regions identified and shared among the PCOS versus control pairwise comparisons at day 0 (red), day 3 (green) and day 12 (blue). Supplemental Fig. 5**.** Screenshots from the UCSC genome browser (hg19) show the overlap between genes *PPARγ* and *CEBPα* and differentially accessible peaks at day 0 and day 12, respectively. Supplemental Fig. 6. Illustration of differentially expressed genes at day 12 that were regulated upstream by *TGFβ1* within the cell nucleus. *TGFβ1* expression was decreased in PCOS versus control cells. Color of bubbles represent relative gene expression levels from our findings, while arrows represent how *TGFβ1* regulates these genes in the literature. Supplemental Fig. 7. Chromatin accessibility and gene expression patterns of candidate genes involved in adipogenesis. Genes involved in (A-B) adipocyte function, (C) androgen action, and (D-F) Wnt signaling are shown. Left panel represents the chromatin accessibility of associated gene regions at days 0, 3, and 12 of adipogenesis. Right panel represents RNA expression levels of each gene at days 0, 3, 7, and 12 of adipogenesis. Values are expressed as mean ± SEM of 3 age and BMI pair-matched normal-weight PCOS and control women. Supplemental Fig. 8. Chromatin accessibility and gene expression patterns of genes involved in the triacylglycerol synthesis functional group. 15 genes involved in the triacylglycerol functional group as determined by Ingenuity Pathway Analysis are shown across adipogenesis. Chromatin accessibility of associated gene regions were analyzed at days 0, 3, and 12 of adipogenesis (first and third panel from the left). RNA expression levels of each gene were analyzed at days 0, 3, 7, and 12 of adipogenesis (second and fourth panel from the left). Values are expressed as mean ± SEM (RGPC for ATAC-seq; CPM for RNA-seq) of 3 age and BMI pair-matched normal-weight PCOS and control women. Supplemental Fig. 9. Total and de novo synthesis of FA fold changes in PCOS versus control at Day 12. Comparison of FA content in newly formed adipocytes from PCOS versus BMI- and age- matched controls for (A) C14:0 myristic acid (B) C16:0 palmitic acid (C) C16:1 palmitoleic acid and (D) C18:1 oleic acid. Values are expressed as fold changes relative to controls. Each color line represents each age- and BMI- pair matched sample.

**Additional file 3:** Additional tables with data from ATAC-seq. Supplemental Table 1. Details of each differentially accessible region at each time point for each patient sample. C and P denotes control and PCOS sample, respectively. Roman numerals denote duplicate samples of each female type. Arabic numerals denote time point. Supplemental Table 2. Results from HOMER transcription factor motif enrichment analysis of differentially accessible regions. Supplemental Table 3. Results from GREAT pathway analysis of differentially accessible regions in PCOS versus control at day 3. Supplemental Table 4. Statistics of ATAC-seq read trimming.

**Additional file 4:** Complete dataset of RNA-seq experiments including counts per million reads (CPM) levels for each sample at each time point and fold-change values between PCOS and control of all genes analyzed. Positive values denote greater expression in PCOS and negative values denote greater expression in controls.

## Data Availability

All sequencing data have been deposited to the NCBI GEO database (https://www.ncbi.nlm.nih.gov/geo/) under accession number GSE156140 for all ATAC-seq-related data and GSE156067 for all RNA-seq-related data.

## Abbreviations

A4: Androstenedione
AP-1: Activator protein-1
AR: Androgen receptor
ASC: Adipose stem cell
ATAC-seq: Assay for transposase-accessible chromatin-sequencing
BMI: Body mass index
ChIP: Chromatin immunoprecipitation
DHEAS: Dehydroepiandrosterone-sulfate
DHT: Dihydrotestosterone
FA: Fatty acid
FCS: Fetal calf serum
FDR: False discovery rate
GC–MS: Gas chromatography-mass spectrometry
GO: Gene ontology
GREAT: Genomic regions enrichment of annotations tool
GSEA: Gene set enrichment analysis
IBMX: 3-Isobutyl-1-methylxanthine
IPA: Ingenuity pathway analysis
ISA: Isotopic spectral analysis
LH: Luteinizing hormone
P4: Progesterone
PCA: Principal component analysis
PCOS: Polycystic ovary syndrome
PCR: Polymerase chain reaction
RNA-seq: RNA-sequencing
SC: Subcutaneous
SVF: Stromal vascular fraction
T: Testosterone
TFBM: Transcription factor binding motifs
TFs: Transcription factors
TMM: Trimmed mean of *M* values
TSS: Transcription start sites

## References

[1] Dumesic DA, Oberfield SE, Stener-Victorin E, Marshall JC, Laven JS, Legro RS (2015). Scientific statement on the diagnostic criteria, epidemiology, pathophysiology, and molecular genetics of polycystic ovary syndrome. Endocr Rev.

[2] Corbould A, Kim Y-B, Youngren JF, Pender C, Kahn BB, Lee A (2005). Insulin resistance in the skeletal muscle of women with PCOS involves intrinsic and acquired defects in insulin signaling. Am J Physiol Endocrinol Metab.

[3] Diamanti-Kandarakis E, Dunaif A (2012). Insulin resistance and the polycystic ovary syndrome revisited: an update on mechanisms and implications. Endocr Rev.

[4] Tosi F, Di Sarra D, Kaufman J-M, Bonin C, Moretta R, Bonora E (2015). Total body fat and central fat mass independently predict insulin resistance but not hyperandrogenemia in women with polycystic ovary syndrome. J Clin Endocrinol Metab.

[5] Dumesic DA, Akopians AL, Madrigal VK, Ramirez E, Margolis DJ, Sarma MK (2016). Hyperandrogenism accompanies increased intra-abdominal fat storage in normal weight polycystic ovary syndrome women. J Clin Endocrinol Metab.

[6] Dumesic DA, Phan JD, Leung KL, Grogan TR, Ding X, Li X (2019). Adipose insulin resistance in normal-weight women with polycystic ovary syndrome. J Clin Endocrinol Metab.

[7] Stener-Victorin E, Padmanabhan V, Walters KA, Campbell RE, Benrick A, Giacobini P (2020). Animal Models to Understand the Etiology and Pathophysiology of Polycystic Ovary Syndrome. Endocr Rev..

[8] Abbott DH, Dumesic DA, Levine JE (2019). Hyperandrogenic origins of polycystic ovary syndrome—implications for pathophysiology and therapy. Expert Rev Endocrinol Metab.

[9] Dumesic DA, Abbott DH, Sanchita S, Chazenbalk GD (2020). Endocrine-metabolic dysfunction in polycystic ovary syndrome: an evolutionary perspective. Curr Opin Endocr Metab Res.

[10] Frayn KN (2002). Adipose tissue as a buffer for daily lipid flux. Diabetologia.

[11] Romacho T, Elsen M, Röhrborn D, Eckel J (2014). Adipose tissue and its role in organ crosstalk. Acta Physiol Oxf Engl.

[12] Chazenbalk G, Singh P, Irge D, Shah A, Abbott DH, Dumesic DA (2013). Androgens inhibit adipogenesis during human adipose stem cell commitment to preadipocyte formation. Steroids.

[13] Cristancho AG, Lazar MA (2011). Forming functional fat: a growing understanding of adipocyte differentiation. Nat Rev Mol Cell Biol.

[14] Tang QQ, Lane MD (2012). Adipogenesis: from stem cell to adipocyte. Annu Rev Biochem.

[15] Corbould A (2007). Chronic testosterone treatment induces selective insulin resistance in subcutaneous adipocytes of women. J Endocrinol.

[16] Arner P (2005). Effects of testosterone on fat cell lipolysis. Species differences and possible role in polycystic ovarian syndrome. Biochimie.

[17] Dicker A, Rydén M, Näslund E, Muehlen IE, Wirén M, Lafontan M (2004). Effect of testosterone on lipolysis in human pre-adipocytes from different fat depots. Diabetologia.

[18] Rosenbaum D, Haber RS, Dunaif A (1993). Insulin resistance in polycystic ovary syndrome: decreased expression of GLUT-4 glucose transporters in adipocytes. Am J Physiol.

[19] Faulds G, Rydén M, Ek I, Wahrenberg H, Arner P (2003). Mechanisms behind lipolytic catecholamine resistance of subcutaneous fat cells in the polycystic ovarian syndrome. J Clin Endocrinol Metab.

[20] Ek I, Arner P, Bergqvist A, Carlström K, Wahrenberg H (1997). Impaired adipocyte lipolysis in nonobese women with the polycystic ovary syndrome: a possible link to insulin resistance?. J Clin Endocrinol Metab.

[21] Spalding KL, Arner E, Westermark PO, Bernard S, Buchholz BA, Bergmann O (2008). Dynamics of fat cell turnover in humans. Nature.

[22] Sinha N, Roy S, Huang B, Wang J, Padmanabhan V, Sen A (2020). Developmental programming: prenatal testosterone-induced epigenetic modulation and its effect on gene expression in sheep ovary. Biol Reprod.

[23] Kokosar M, Benrick A, Perfilyev A, Fornes R, Nilsson E, Maliqueo M (2016). Epigenetic and transcriptional alterations in human adipose tissue of polycystic ovary syndrome. Sci Rep.

[24] Farmer SR (2006). Transcriptional control of adipocyte formation. Cell Metab.

[25] Fisch SC, Nikou AF, Wright EA, Phan JD, Leung KL, Grogan TR (2018). Precocious subcutaneous abdominal stem cell development to adipocytes in normal-weight women with polycystic ovary syndrome. Fertil Steril.

[26] Rosen ED, MacDougald OA (2006). Adipocyte differentiation from the inside out. Nat Rev Mol Cell Biol.

[27] Rosen ED, Hsu C-H, Wang X, Sakai S, Freeman MW, Gonzalez FJ (2002). C/EBPα induces adipogenesis through PPARγ: a unified pathway. Genes Dev.

[28] Siersbæk R, Mandrup S (2011). Transcriptional networks controlling adipocyte differentiation. Cold Spring Harb Symp Quant Biol.

[29] Salma N, Xiao H, Imbalzano AN (2020). Temporal recruitment of CCAAT/enhancer-binding proteins to early and late adipogenic promoters in vivo. J Mol Endocrinol.

[30] Siersbæk R, Nielsen R, Mandrup S (2012). Transcriptional networks and chromatin remodeling controlling adipogenesis. Trends Endocrinol Metab.

[31] Siersbæk R, Madsen JGS, Javierre BM, Nielsen R, Bagge EK, Cairns J (2017). Dynamic rewiring of promoter-anchored chromatin loops during adipocyte differentiation. Mol Cell.

[32] Stephens JM (2012). The fat controller: adipocyte development. PLoS Biol.

[33] Moitra J, Mason MM, Olive M, Krylov D, Gavrilova O, Marcus-Samuels B (1998). Life without white fat: a transgenic mouse. Genes Dev.

[34] Lefterova MI, Haakonsson AK, Lazar MA, Mandrup S (2014). PPARγ and the global map of adipogenesis and beyond. Trends Endocrinol Metab TEM.

[35] Pellegrinelli V, Carobbio S, Vidal-Puig A (2016). Adipose tissue plasticity: how fat depots respond differently to pathophysiological cues. Diabetologia.

[36] Chawla A, Nguyen KD, Goh YPS (2011). Macrophage-mediated inflammation in metabolic disease. Nat Rev Immunol.

[37] Chazenbalk G, Bertolotto C, Heneidi S, Jumabay M, Trivax B, Aronowitz J (2011). Novel pathway of adipogenesis through cross-talk between adipose tissue macrophages, adipose stem cells and adipocytes: evidence of cell plasticity. PLoS ONE.

[38] Jones MR, Chazenbalk G, Xu N, Chua AK, Eigler T, Mengesha E (2012). Steroidogenic regulatory factor FOS is underexpressed in polycystic ovary syndrome (PCOS) adipose tissue and genetically associated with PCOS susceptibility. J Clin Endocrinol Metab.

[39] Kalluri R, LeBleu VS. The biology, function, and biomedical applications of exosomes. Science [Internet]. 2020 Feb 7 [cited 2020 Aug 16]; 367(6478). https://science.sciencemag.org/content/367/6478/eaau6977 10.1126/science.aau6977 PMC7717626 32029601

[40] Qi X, Zhang J, Yuan H, Xu Z, Li Q, Niu X (2016). Exosomes secreted by human-induced pluripotent stem cell-derived mesenchymal stem cells repair critical-sized bone defects through enhanced angiogenesis and osteogenesis in osteoporotic rats. Int J Biol Sci.

[41] Wang X, Omar O, Vazirisani F, Thomsen P, Ekström K (2018). Mesenchymal stem cell-derived exosomes have altered microRNA profiles and induce osteogenic differentiation depending on the stage of differentiation. PLoS ONE.

[42] Xu N, Kwon S, Abbott DH, Geller DH, Dumesic DA, Azziz R (2011). Epigenetic mechanism underlying the development of polycystic ovary syndrome (PCOS)-like phenotypes in prenatally androgenized rhesus monkeys. PLoS ONE.

[43] Xu J, Bao X, Peng Z, Wang L, Du L, Niu W (2016). Comprehensive analysis of genome-wide DNA methylation across human polycystic ovary syndrome ovary granulosa cell. Oncotarget.

[44] Almodóvar-García K, Kwon M, Samaras SE, Davidson JM (2014). ANKRD1 Acts as a transcriptional repressor of MMP13 via the AP-1 site. Mol Cell Biol.

[45] Fernández-Galilea M, Tapia P, Cautivo K, Morselli E, Cortés VA (2015). AGPAT2 deficiency impairs adipogenic differentiation in primary cultured preadipocytes in a non-autophagy or apoptosis dependent mechanism. Biochem Biophys Res Commun.

[46] Kennell JA, MacDougald OA (2005). Wnt signaling inhibits adipogenesis through beta-catenin-dependent and -independent mechanisms. J Biol Chem.

[47] Bennett CN, Ross SE, Longo KA, Bajnok L, Hemati N, Johnson KW (2002). Regulation of Wnt signaling during adipogenesis. J Biol Chem.

[48] Choy L, Derynck R (2003). Transforming growth factor-beta inhibits adipocyte differentiation by Smad3 interacting with CCAAT/enhancer-binding protein (C/EBP) and repressing C/EBP transactivation function. J Biol Chem.

[49] Tsurutani Y, Fujimoto M, Takemoto M, Irisuna H, Koshizaka M, Onishi S (2011). The roles of transforming growth factor-β and Smad3 signaling in adipocyte differentiation and obesity. Biochem Biophys Res Commun.

[50] Raja-Khan N, Urbanek M, Rodgers RJ, Legro RS (2014). The role of TGF-β in polycystic ovary syndrome. Reprod Sci Thousand Oaks Calif.

[51] Li S-N, Wu J-F (2020). TGF-β/SMAD signaling regulation of mesenchymal stem cells in adipocyte commitment. Stem Cell Res Ther.

[52] Martínez L, Torres S, Baulies A, Alarcón-Vila C, Elena M, Fabriàs G (2015). Myristic acid potentiates palmitic acid-induced lipotoxicity and steatohepatitis associated with lipodystrophy by sustaning de novo ceramide synthesis. Oncotarget.

[53] Acosta-Montaño P, Rodríguez-Velázquez E, Ibarra-López E, Frayde-Gómez H, Mas-Oliva J, Delgado-Coello B, et al. Fatty acid and lipopolysaccharide effect on beta cells proteostasis and its impact on insulin secretion. Cells [Internet]. 2019 Aug 13 [cited 2020 Aug 15]; 8(8). https://www.ncbi.nlm.nih.gov/pmc/articles/PMC6721695/10.3390/cells8080884 PMC6721695 31412623

[54] Listenberger LL, Han X, Lewis SE, Cases S, Farese RV, Ory DS (2003). Triglyceride accumulation protects against fatty acid-induced lipotoxicity. Proc Natl Acad Sci U S A.

[55] Frankel EN (1984). Lipid oxidation: mechanisms, products and biological significance. J Am Oil Chem Soc.

[56] Fessler DMT, Natterson-Horowitz B, Azziz R (2016). Evolutionary determinants of polycystic ovary syndrome: part 2. Fertil Steril.

[57] Virtue S, Vidal-Puig A (2010). Adipose tissue expandability, lipotoxicity and the metabolic syndrome—an allostatic perspective. Biochim Biophys Acta.

[58] Keller E, Chazenbalk GD, Aguilera P, Madrigal V, Grogan T, Elashoff D (2014). Impaired preadipocyte differentiation into adipocytes in subcutaneous abdominal adipose of PCOS-like female rhesus monkeys. Endocrinology.

[59] Padmanabhan V, Veiga-Lopez A (2013). Sheep models of polycystic ovary syndrome phenotype. Mol Cell Endocrinol.

[60] Buenrostro JD, Wu B, Chang HY, Greenleaf WJ (2015). ATAC-seq: a method for assaying chromatin accessibility genome-wide. Curr Protoc Mol Biol.

[61] Andrews S. Babraham bioinformatics—FastQC a quality control tool for high throughput sequence data [Internet]. [cited 2020 Aug 16]. https://www.bioinformatics.babraham.ac.uk/projects/fastqc/

[62] Bolger AM, Lohse M, Usadel B (2014). Trimmomatic: a flexible trimmer for Illumina sequence data. Bioinforma Oxf Engl.

[63] Langmead B, Salzberg SL (2012). Fast gapped-read alignment with Bowtie 2. Nat Methods.

[64] Broad Institute. Picard Tools—By Broad Institute [Internet]. [cited 2020 Aug 16]. https://broadinstitute.github.io/picard/

[65] Ramírez F, Ryan DP, Grüning B, Bhardwaj V, Kilpert F, Richter AS (2016). deepTools2: a next generation web server for deep-sequencing data analysis. Nucl Acids Res.

[66] Zhang Y, Liu T, Meyer CA, Eeckhoute J, Johnson DS, Bernstein BE (2008). Model-based analysis of ChIP-Seq (MACS). Genome Biol.

[67] Stark R, Brown G. DiffBind: differential binding analysis of ChIP-Seq peak data. 2011;33

[68] Yu G, Wang L-G, He Q-Y (2015). ChIPseeker: an R/Bioconductor package for ChIP peak annotation, comparison and visualization. Bioinform Oxf Engl.

[69] McLean CY, Bristor D, Hiller M, Clarke SL, Schaar BT, Lowe CB (2010). GREAT improves functional interpretation of cis-regulatory regions. Nat Biotechnol.

[70] Li B, Dewey CN (2011). RSEM: accurate transcript quantification from RNA-Seq data with or without a reference genome. BMC Bioinform.

[71] Robinson MD, McCarthy DJ, Smyth GK (2010). edgeR: a Bioconductor package for differential expression analysis of digital gene expression data. Bioinform Oxf Engl.

[72] Love MI, Huber W, Anders S (2014). Moderated estimation of fold change and dispersion for RNA-seq data with DESeq2. Genome Biol.

[73] Subramanian A, Tamayo P, Mootha VK, Mukherjee S, Ebert BL, Gillette MA (2005). Gene set enrichment analysis: a knowledge-based approach for interpreting genome-wide expression profiles. Proc Natl Acad Sci U S A.

[74] Liberzon A, Birger C, Thorvaldsdóttir H, Ghandi M, Mesirov JP, Tamayo P (2015). The Molecular Signatures Database (MSigDB) hallmark gene set collection. Cell Syst.

[75] Argus JP, Wilks MQ, Zhou QD, Hsieh WY, Khialeeva E, Hoi XP (2018). Development and application of FASA, a model for quantifying fatty acid metabolism using stable isotope labeling. Cell Rep..

