# Supplementary figures and images for: Dynamic changes in chromatin accessibility, altered adipogenic gene expression, and total versus de novo fatty acid synthesis in subcutaneous adipose stem cells of normal-weight polycystic ovary syndrome (PCOS) women during adipogenesis: evidence of cellular programming
Source: Clin Epigenetics. 2020 Nov 23;12:181. doi: 10.1186/s13148-020-00970-x (PMC7686698; doi:10.1186/s13148-020-00970-x)

## Slide 1
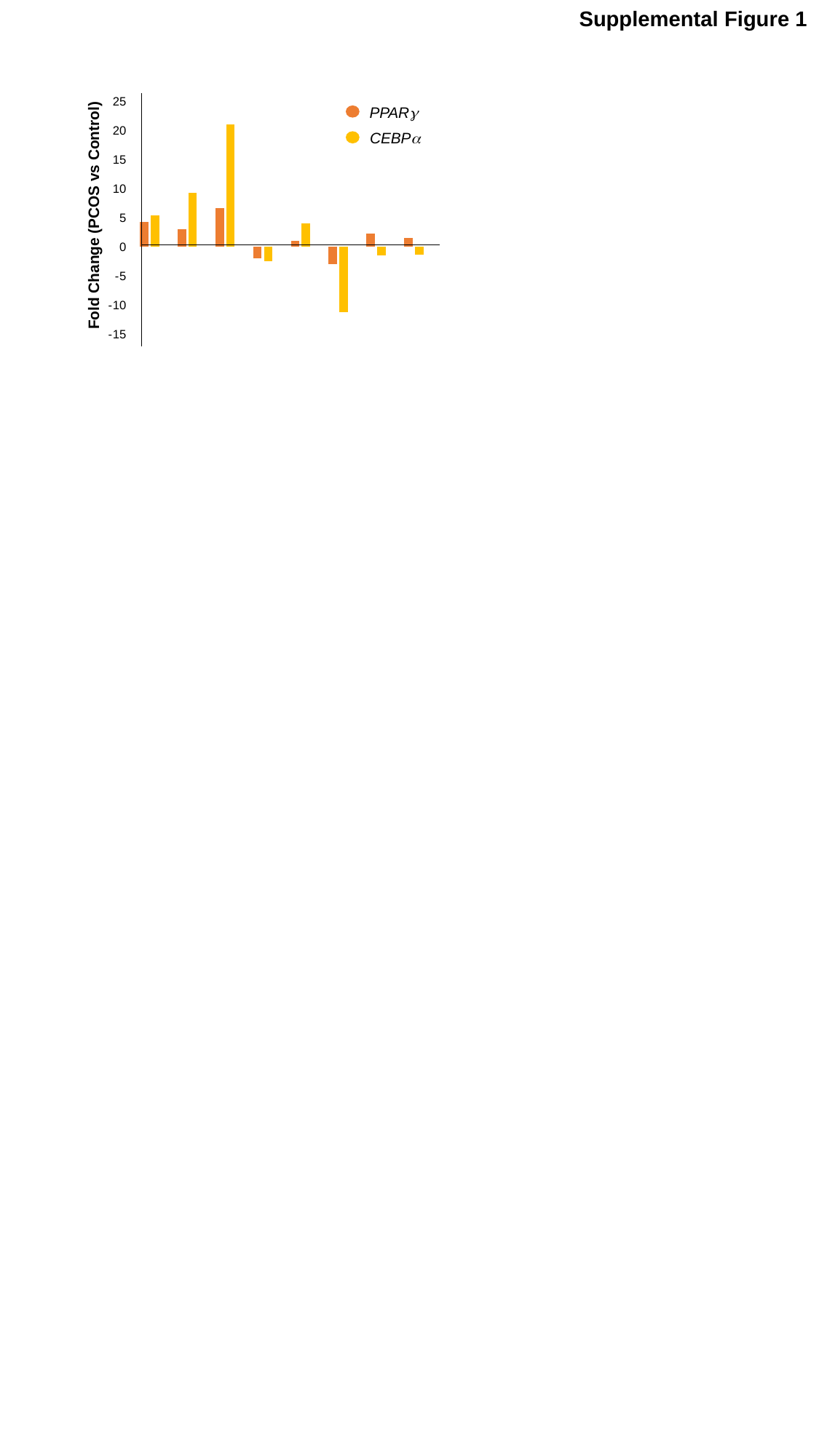

Supplemental Figure 1
### Chart
| Category | | |
|---|---|---|PPARg
CEBPa
Fold Change (PCOS vs Control)

Supplement: Supplementary file 1 — Additional file 1: Additional figure of PPARγ and CEBPα expression of 8 PCOS versus 8 age- and BMI-matched control women from our previous study (Fisch, 2018). Supplemental Fig. 1. Fold changes of PPARγ and CEBPα gene expression levels at day 12 of adipogenesis. Values represent PCOS versus control. Three out of eight total pair match subjects were selected for the present study based on significantly higher (> 2.0-fold) gene expression levels of PPARγ and CEBPα in PCOS. [file 13148_2020_970_MOESM1_ESM.pptx]
