# Supplementary material for: Dynamic changes in chromatin accessibility, altered adipogenic gene expression, and total versus de novo fatty acid synthesis in subcutaneous adipose stem cells of normal-weight polycystic ovary syndrome (PCOS) women during adipogenesis: evidence of cellular programming
Source: Clin Epigenetics. 2020 Nov 23;12:181. doi: 10.1186/s13148-020-00970-x (PMC7686698; doi:10.1186/s13148-020-00970-x)
Supplement: Supplementary file 2 — Additional file 2: Additional figures with data from ATAC-seq and RNA-seq. Supplemental Fig. 2. PCA plots from 3 PCOS and 3 control samples of (A) ATAC-seq data at days 0, 3, and 12 of adipogenesis and (B) RNA-seq data at days 0, 3, 7, and 12 of adipogenesis. Time points are labeled using different colors while female-type is labeled using different shapes. Supplemental Fig. 3. Heatmaps of global chromatin accessibility changes at gene transcription start sites (TSS) in PCOS and control samples across time. ATAC-seq read density in each sample within a 6 kb window centered at all annotated TSS in the genome are shown from high (blue) to low (red) read densities. Higher ATAC-seq read density at TSS (e.g. day 3) suggests higher chromatin accessibility. Above each heatmap, a profile plots summarizes the normalized Reads Per Genome Content (RPGC) across all TSS. Supplemental Fig. 4. Venn diagram of the number of significant differentially accessible regions identified and shared among the PCOS versus control pairwise comparisons at day 0 (red), day 3 (green) and day 12 (blue). Supplemental Fig. 5. Screenshots from the UCSC genome browser (hg19) show the overlap between genes PPARγ and CEBPα and differentially accessible peaks at day 0 and day 12, respectively. Supplemental Fig. 6. Illustration of differentially expressed genes at day 12 that were regulated upstream by TGFβ1 within the cell nucleus. TGFβ1 expression was decreased in PCOS versus control cells. Color of bubbles represent relative gene expression levels from our findings, while arrows represent how TGFβ1 regulates these genes in the literature. Supplemental Fig. 7. Chromatin accessibility and gene expression patterns of candidate genes involved in adipogenesis. Genes involved in (A-B) adipocyte function, (C) androgen action, and (D-F) Wnt signaling are shown. Left panel represents the chromatin accessibility of associated gene regions at days 0, 3, and 12 of adipogenesis. Right panel represents RNA expressi [file 13148_2020_970_MOESM2_ESM.pptx]

## Slide 1
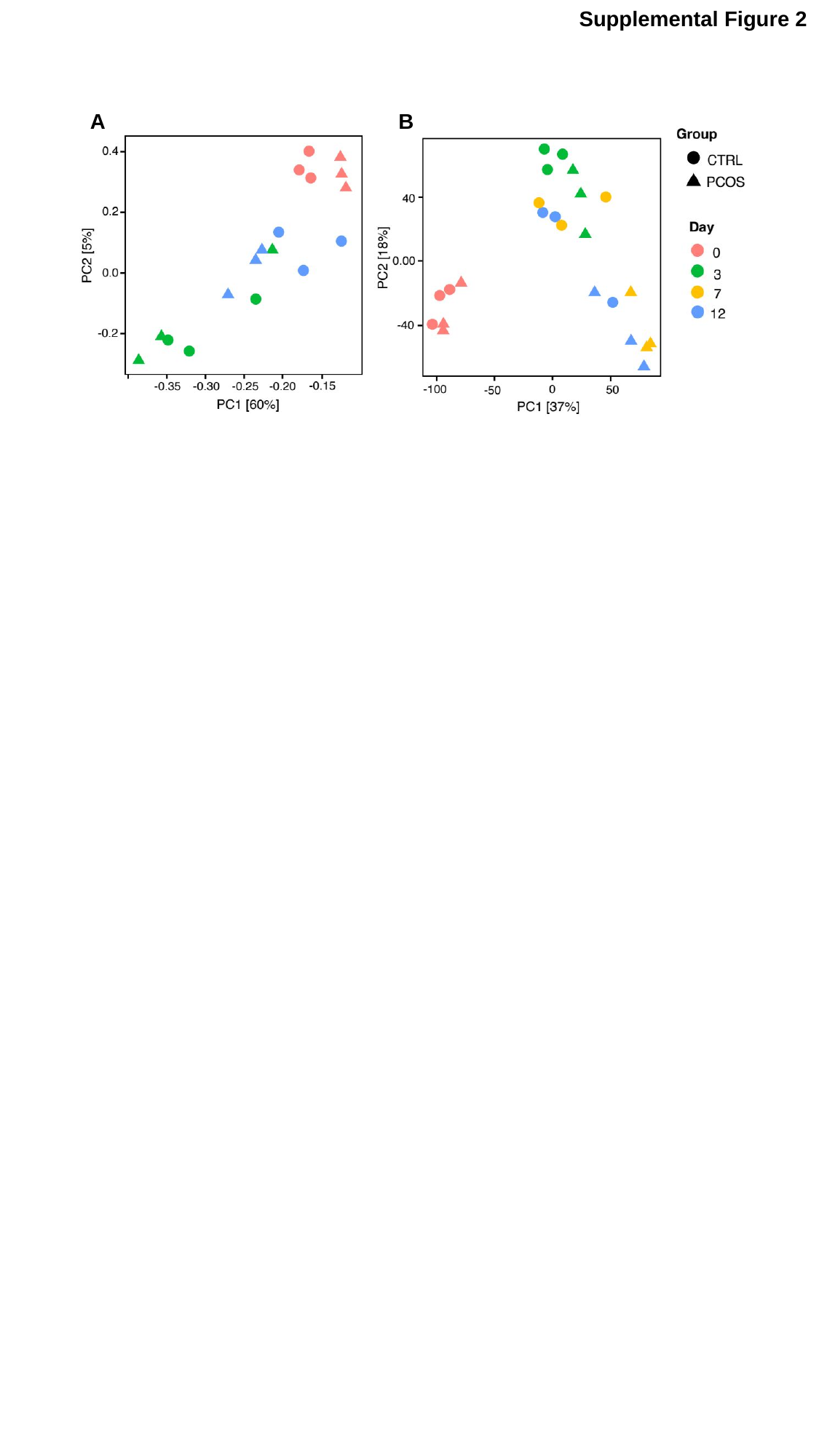

Supplemental Figure 2
A
B

## Slide 2
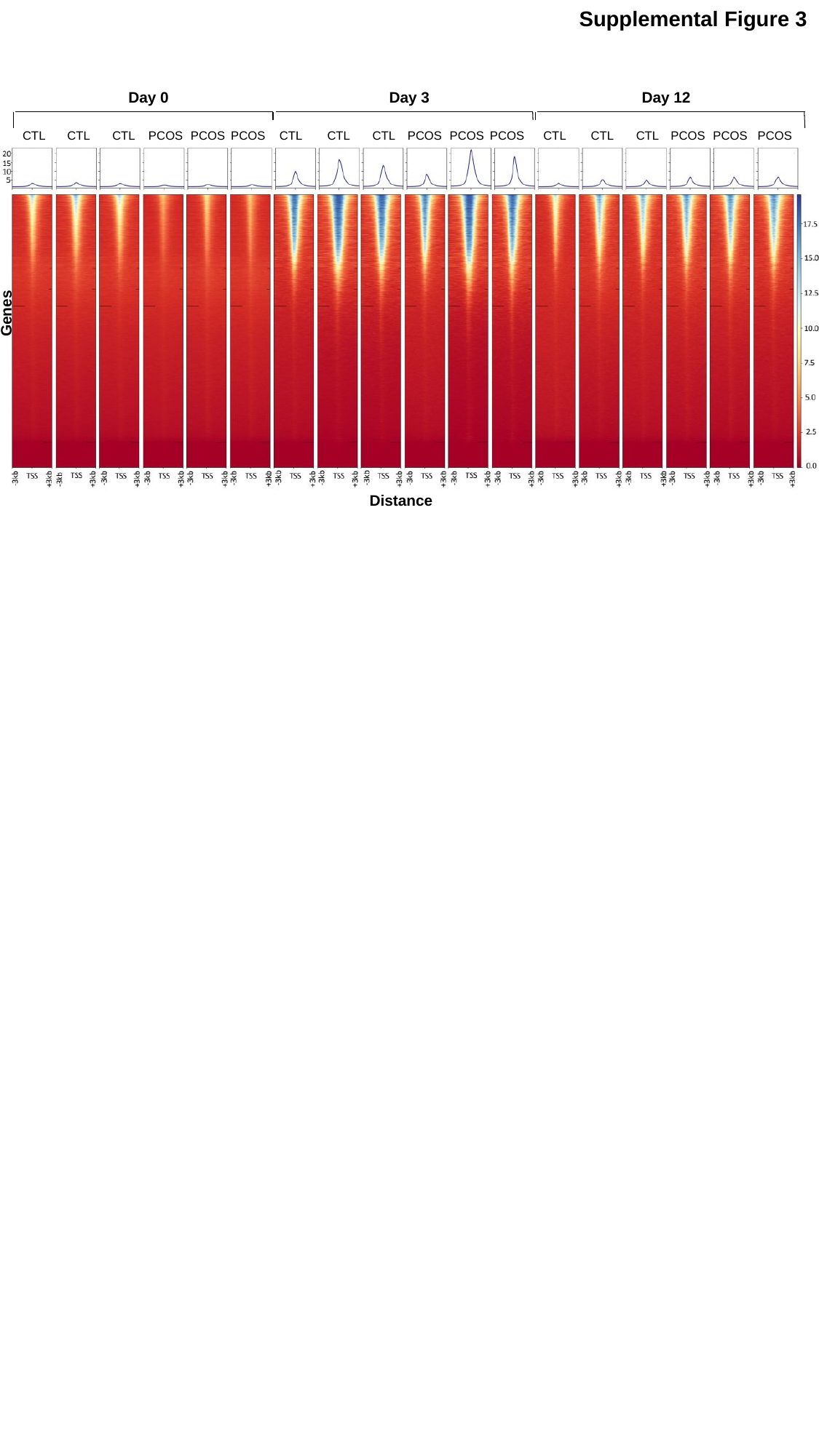

Supplemental Figure 3
Day 0
Day 3
Day 12
CTL
CTL
CTL
PCOS
PCOS
PCOS
CTL
CTL
CTL
PCOS
PCOS
PCOS
CTL
CTL
CTL
PCOS
PCOS
PCOS
Genes
Distance

## Slide 3
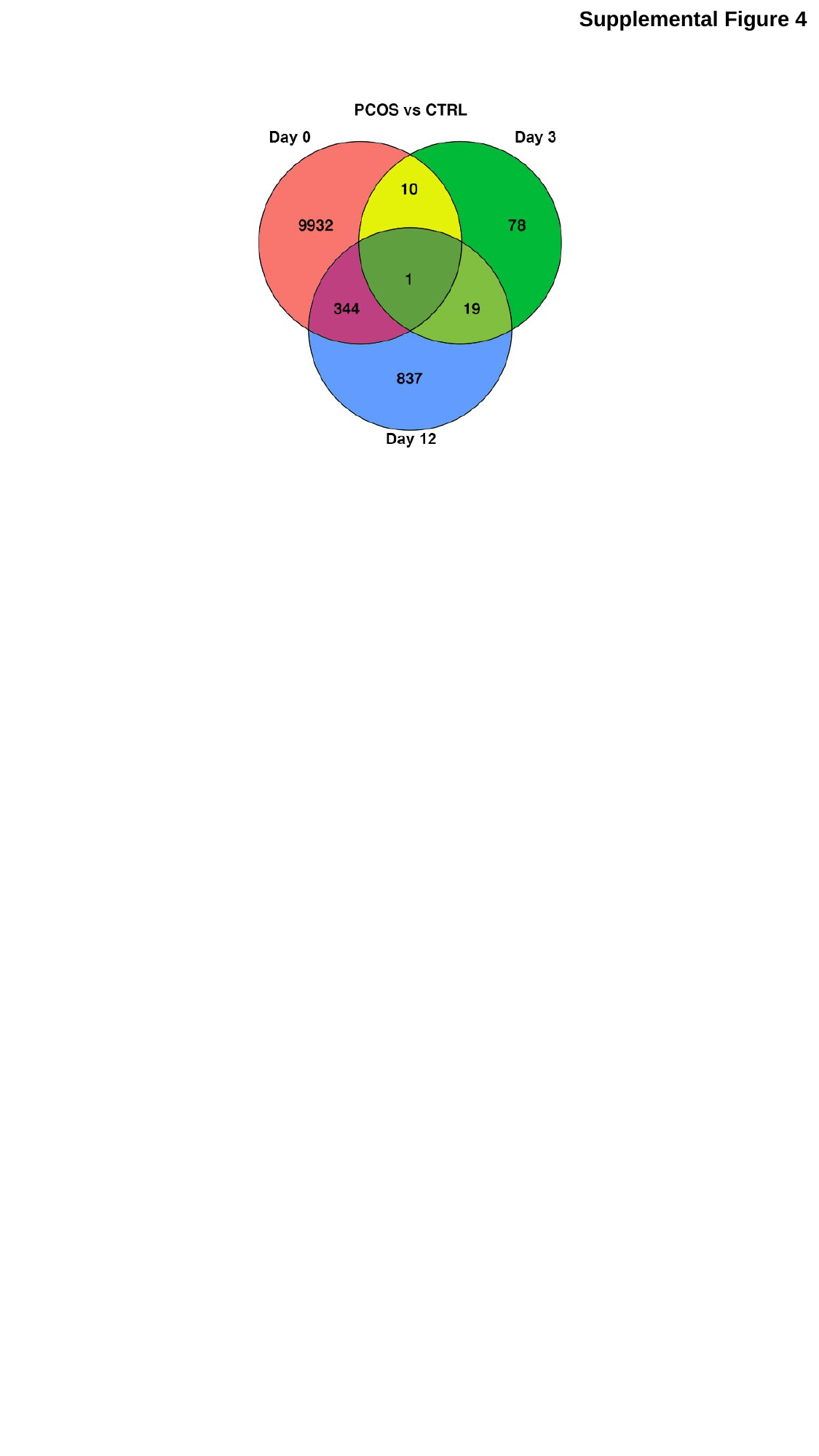

Supplemental Figure 4

## Slide 4
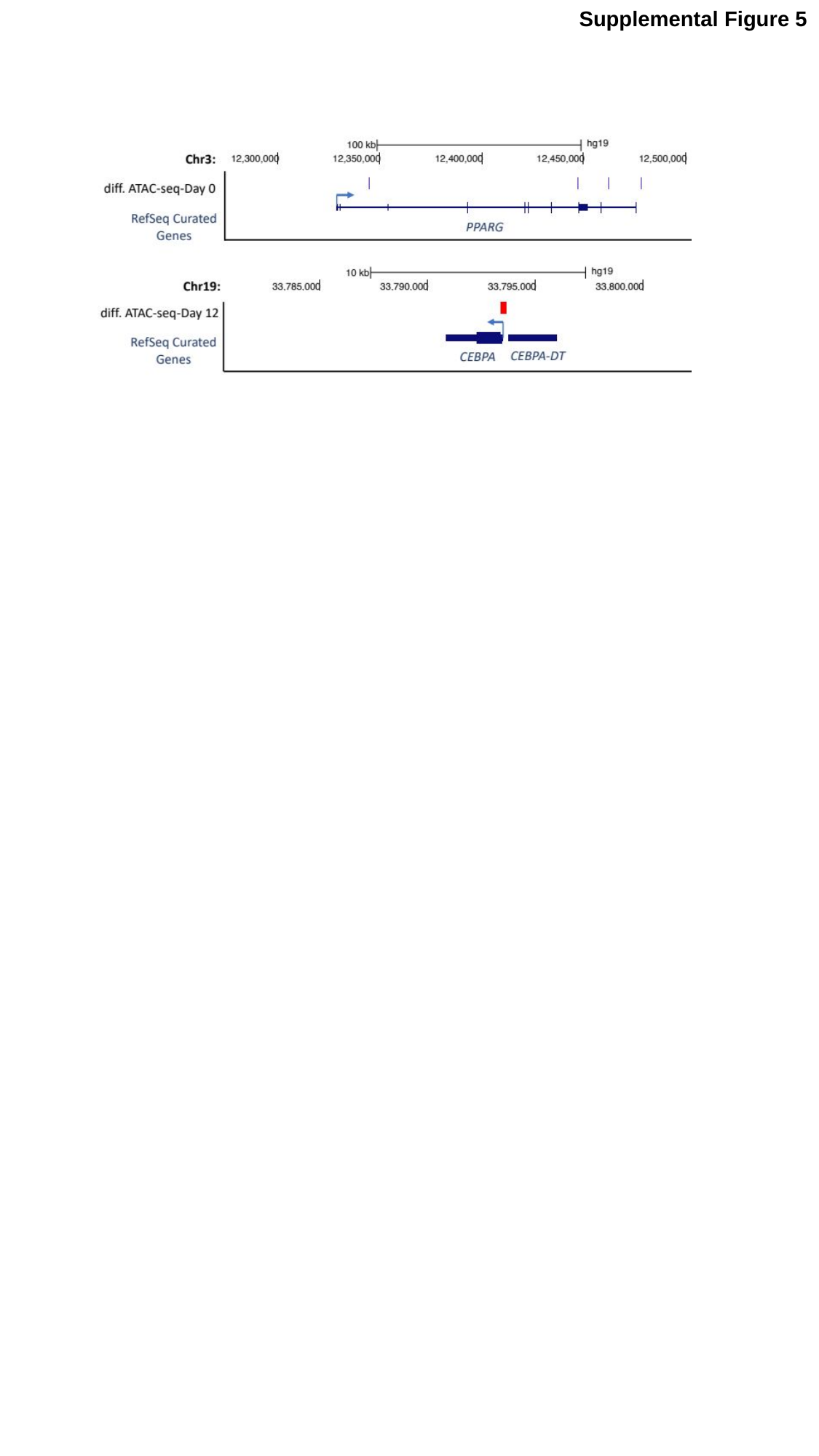

Supplemental Figure 5

## Slide 5
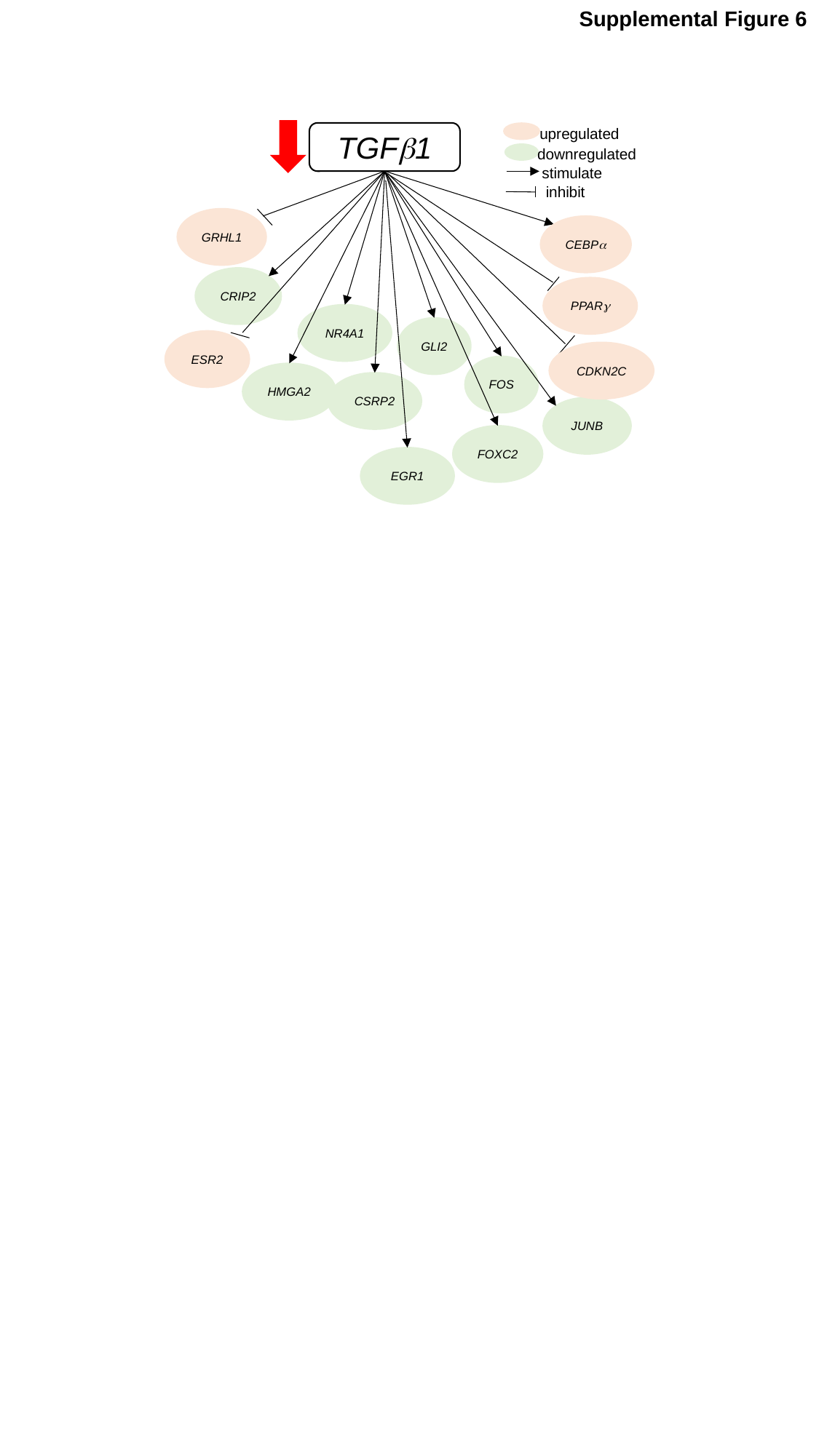

Supplemental Figure 6
upregulated
downregulated
stimulate
inhibit
TGFb1
GRHL1
CEBPa
CRIP2
PPARg
NR4A1
GLI2
ESR2
CDKN2C
FOS
HMGA2
CSRP2
JUNB
FOXC2
EGR1

## Slide 6
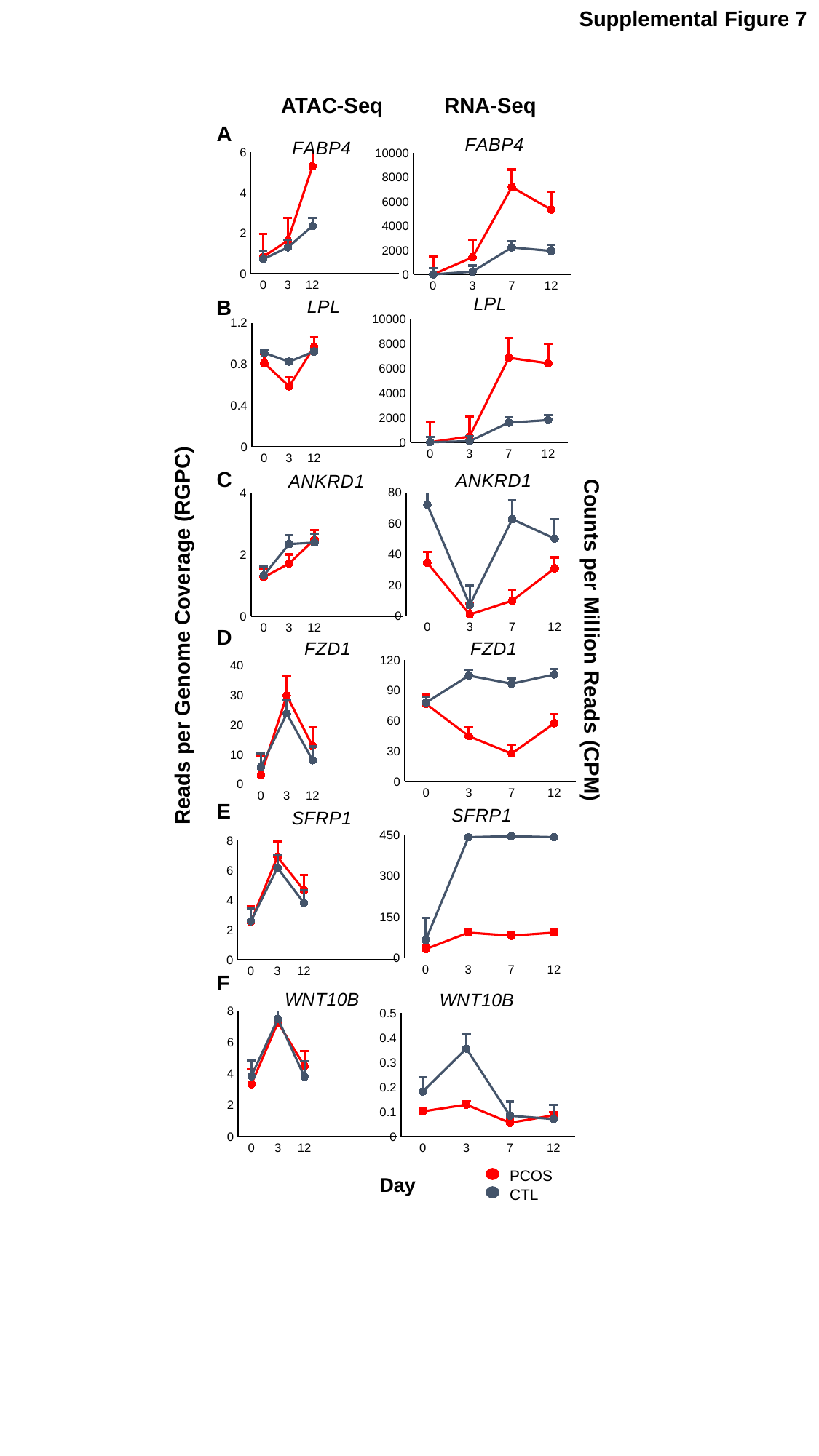

Supplemental Figure 7
ATAC-Seq
RNA-Seq
[unsupported chart]
### Chart: FABP4
| Category | | |
|---|---|---|
| 0 | 1.387866333 | 2.0043152416666667 |
| 3 | 1410.0622534333334 | 232.2373750733333 |
| 7 | 7187.8663203333335 | 2217.7892003 |
| 12 | 5335.027918333333 | 1934.4873219 |A
B
C
D
E
F
### Chart: LPL
| Category | | |
|---|---|---|
| 0 | 1.6145949553333334 | 2.0352284276666666 |
| 3 | 457.4852067 | 82.27446843333333 |
| 7 | 6837.150850666668 | 1583.9976465666666 |
| 12 | 6392.428618333334 | 1793.5703530666667 |
[unsupported chart]
### Chart: ANKRD1
| Category | | |
|---|---|---|
| 0 | 34.372888716666665 | 71.99073103666667 |
| 3 | 0.8169174193333334 | 7.060310801666667 |
| 7 | 9.776678959666667 | 62.58556186666667 |
| 12 | 30.809544416666668 | 50.06458740666667 |
[unsupported chart]
### Chart: FZD1
| Category | | |
|---|---|---|
| 0 | 76.56282809666665 | 78.04816076 |
| 3 | 44.65829277666666 | 104.52113179666667 |
| 7 | 27.4429785 | 96.55082804666667 |
| 12 | 57.46368905333333 | 105.68247392666666 |
[unsupported chart]
Reads per Genome Coverage (RGPC)
Counts per Million Reads (CPM)
### Chart: SFRP1
| Category | | |
|---|---|---|
| 0 | 32.33179410666667 | 65.14847624333333 |
| 3 | 92.20062090333333 | 440.1975364 |
| 7 | 81.27283959666666 | 443.87138513333326 |
| 12 | 92.20062090333333 | 440.1975364 |
[unsupported chart]
[unsupported chart]
### Chart: WNT10B
| Category | | |
|---|---|---|
| 0 | 0.101481491 | 0.182065165 |
| 3 | 0.12914323266666666 | 0.35645940400000004 |
| 7 | 0.05562613166666667 | 0.084249236 |
| 12 | 0.08598866933333332 | 0.07067459833333334 |PCOS
CTL
Day

## Slide 7
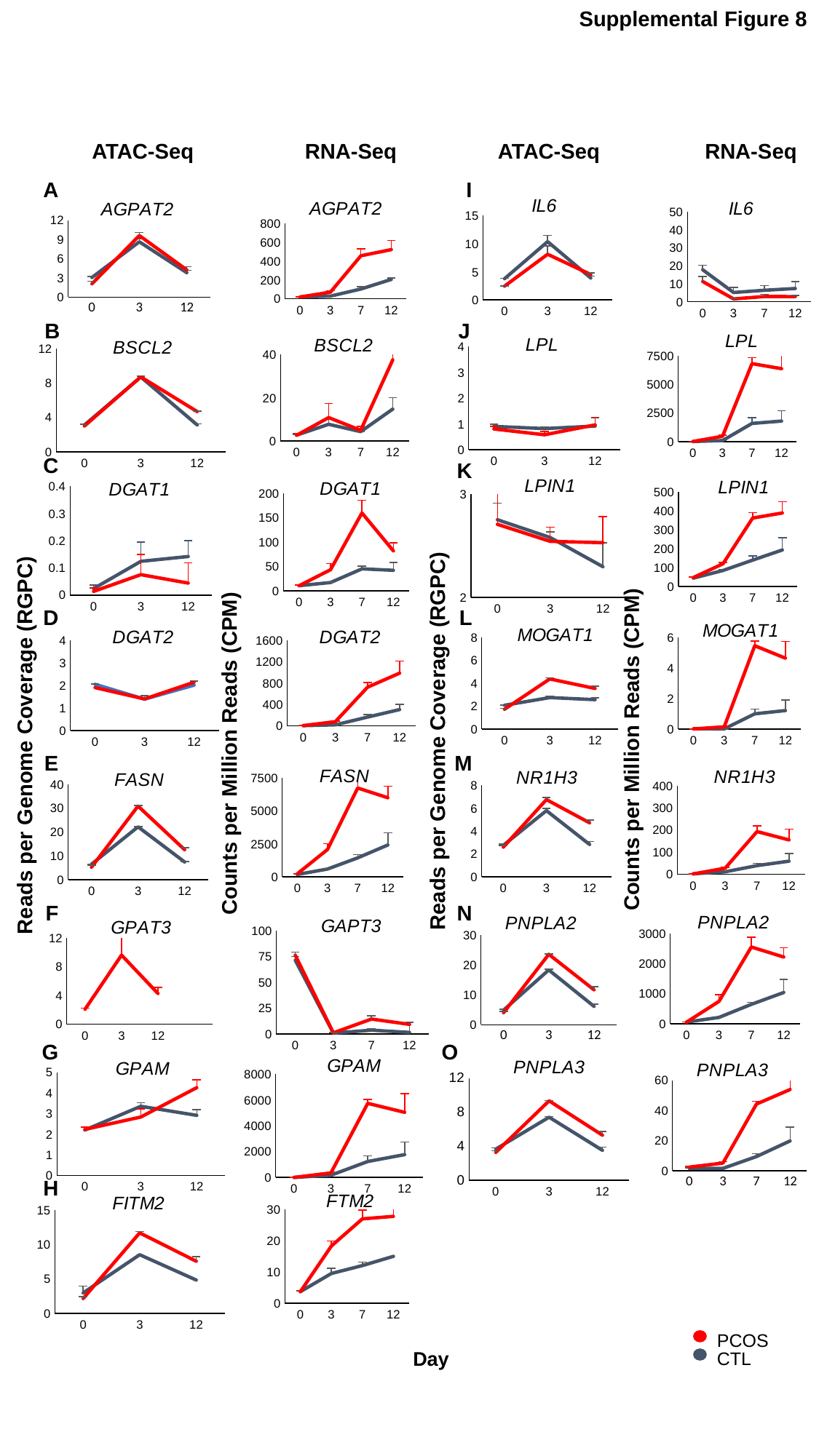

Supplemental Figure 8
ATAC-Seq
RNA-Seq
ATAC-Seq
RNA-Seq
### Chart: IL6
| Category | Cotrol | PCOS |
|---|---|---|
| 0 | 3.7764174258424865 | 2.4511358333794036 |
| 3 | 10.36184410857385 | 8.11640807184212 |
| 12 | 3.89458658499816 | 4.524995720529526 |
### Chart: IL6
| Category | Control | PCOS |
|---|---|---|
| 0 | 17.768752503333335 | 11.237441917666667 |
| 3 | 5.103980809 | 1.542594842 |
| 7 | 6.358039377 | 2.8723816056666664 |
| 12 | 7.327856407666666 | 2.826221420333333 |A
I
### Chart: AGPAT2
| Category | Control | PCOS |
|---|---|---|
| 0 | 18.628988949999997 | 17.97598422 |
| 3 | 27.583351046666664 | 70.16892411333333 |
| 7 | 102.83394425333334 | 455.9372849666667 |
| 12 | 204.87808081000003 | 521.0653556666666 |
### Chart: AGPAT2
| Category | Cotrol | PCOS |
|---|---|---|
| 0 | 3.0622536124374804 | 2.06965855874929 |
| 3 | 8.628319292028932 | 9.59198613990341 |
| 12 | 3.814540006583283 | 4.25283339136919 |
### Chart: LPL
| Category | Cotrol | PCOS |
|---|---|---|
| 0 | 0.9080511981902056 | 0.8084000117602107 |
| 3 | 0.8227666464175326 | 0.5831194157693896 |
| 12 | 0.9208653509246036 | 0.9662091068735951 |
### Chart: BSCL2
| Category | Control | PCOS |
|---|---|---|
| 0 | 3.104340848939677 | 2.9881822273382634 |
| 3 | 8.695292622875664 | 8.705018857058693 |
| 12 | 3.1156989943614968 | 4.66697711045825 |
### Chart: LPL
| Category | Control | PCOS |
|---|---|---|
| 0 | 2.0352284276666666 | 1.6145949553333334 |
| 3 | 82.27446843333333 | 457.4852067 |
| 7 | 1583.9976465666666 | 6837.150850666668 |
| 12 | 1793.5703530666667 | 6392.428618333334 |B
J
### Chart: BSCL2
| Category | Control | PCOS |
|---|---|---|
| 0 | 2.7804279116666666 | 2.7018803676666665 |
| 3 | 7.777990996000001 | 10.889013588333333 |
| 7 | 4.374488817333334 | 5.027220662666666 |
| 12 | 14.840679043666666 | 37.62578769666667 |
### Chart: LPIN1
| Category | Control | PCOS |
|---|---|---|
| 0 | 43.93467592666667 | 47.28244513333333 |
| 3 | 84.52913585 | 119.9004897 |
| 7 | 139.28608646666666 | 362.3591681 |
| 12 | 192.94467324666664 | 389.02267266666667 |
### Chart: LPIN1
| Category | Control | PCOS |
|---|---|---|
| 0 | 2.7540493324909634 | 2.709142276169763 |
| 3 | 2.58106722497929 | 2.54390684390461 |
| 12 | 2.298186866700933 | 2.5312195100887163 |C
### Chart: DGAT1
| Category | Control | PCOS |
|---|---|---|
| 0 | 10.298746665333333 | 10.142550228333333 |
| 3 | 16.816381926666665 | 43.09681743000001 |
| 7 | 44.81879946666667 | 159.6567639333333 |
| 12 | 41.839762146666665 | 81.61969696333334 |K
### Chart: DGAT1
| Category | Cotrol | PCOS |
|---|---|---|
| 0 | 0.023532893196421236 | 0.0131409888544831 |
| 3 | 0.12386959535919424 | 0.07492059089472267 |
| 12 | 0.14169255913955972 | 0.04366588024393033 |
### Chart: MOGAT1
| Category | Cotrol | PCOS |
|---|---|---|
| 0 | 2.06928613786067 | 1.72183385293384 |
| 3 | 2.7380570751166666 | 4.3640158532897795 |
| 12 | 2.5588516804739267 | 3.5327710632131493 |
### Chart: DGAT2
| Category | Control | PCOS |
|---|---|---|
| 0 | 2.050615161238507 | 1.90554217581933 |
| 3 | 1.3941359743206867 | 1.4018060698420698 |
| 12 | 2.0134488806730197 | 2.1432839567440234 |
### Chart: MOGAT1
| Category | PCOS | |
|---|---|---|
| 0 | 0.010666666666666666 | 0.0 |
| 3 | 0.0 | 0.13455840866666666 |
| 7 | 0.9897585433333335 | 5.471485948000001 |
| 12 | 1.2044481703333334 | 4.649868892 |
### Chart: DGAT2
| Category | Control | PCOS |
|---|---|---|
| 0 | 3.8958010323333334 | 4.758075553333334 |
| 3 | 18.600704876666665 | 77.98975516333334 |
| 7 | 163.05232716666666 | 721.0130074666667 |
| 12 | 303.54847083333334 | 985.6672540333334 |D
L
Counts per Million Reads (CPM)
Counts per Million Reads (CPM)
### Chart: NR1H3
| Category | Control | PCOS |
|---|---|---|
| 0 | 2.75252422424698 | 2.59510063859864 |
| 3 | 5.7920889697895666 | 6.762383923539581 |
| 12 | 2.8341595790044867 | 4.726704190068787 |Reads per Genome Coverage (RGPC)
Reads per Genome Coverage (RGPC)
### Chart: FASN
| Category | Control | PCOS |
|---|---|---|
| 0 | 178.3798392 | 229.0038547 |
| 3 | 587.1010501666666 | 2065.251474 |
| 7 | 1435.375823333333 | 6731.1225933333335 |
| 12 | 2410.946155 | 5990.258007333334 |
### Chart: NR1H3
| Category | Control | PCOS |
|---|---|---|
| 0 | 1.8378426333333333 | 1.2218937116666666 |
| 3 | 11.066524145 | 27.328050603333335 |
| 7 | 38.96111466 | 192.99485763333334 |
| 12 | 58.952751093333326 | 155.16601039333332 |
### Chart: FASN
| Category | Control | PCOS |
|---|---|---|
| 0 | 6.444614975239067 | 5.193399725162677 |
| 3 | 22.1531118363703 | 30.8771294165285 |
| 12 | 7.3925800653442195 | 12.5299661929214 |E
M
### Chart: PNPLA2
| Category | Control | PCOS |
|---|---|---|
| 0 | 4.922850396867154 | 4.056756968883074 |
| 3 | 18.336454351325866 | 23.58018889610533 |
| 12 | 6.1617062505447775 | 11.688317200590939 |
### Chart: GAPT3
| Category | Control | PCOS |
|---|---|---|
| 0 | 71.31820768 | 76.12575169666667 |
| 3 | 0.7008536006666666 | 0.921275709 |
| 7 | 3.5013444103333335 | 14.298739383000003 |
| 12 | 1.3656718426666667 | 9.145726867666667 |
### Chart: GPAT3
| Category | Control | PCOS |
|---|---|---|
| 0 | 2.19628302067549 | 2.06965855874929 |
| 3 | 8.628319292028932 | 9.59198613990341 |
| 12 | 3.814540006583283 | 4.25283339136919 |F
N
### Chart: PNPLA2
| Category | Control | PCOS |
|---|---|---|
| 0 | 54.04471090333334 | 51.07344771666667 |
| 3 | 207.25168793333333 | 742.2864473333333 |
| 7 | 643.5804092 | 2550.341172 |
| 12 | 1038.0135057 | 2216.709032 |
### Chart: PNPLA3
| Category | Control | PCOS |
|---|---|---|
| 0 | 1.6592756069999999 | 2.466424226333333 |
| 3 | 1.6463331529999998 | 5.153835253333333 |
| 7 | 9.467543915999999 | 44.25661198 |
| 12 | 19.814883048 | 53.85750551666666 |
### Chart: GPAM
| Category | Control | PCOS |
|---|---|---|
| 0 | 2.19628302067549 | 2.2283413716194533 |
| 3 | 3.349094888965053 | 2.8265750057457666 |
| 12 | 2.9079920337997103 | 4.252867632415007 |
### Chart: GPAM
| Category | Control | PCOS |
|---|---|---|
| 0 | 2.419031429 | 2.534328463 |
| 3 | 169.81033806666665 | 349.7835344 |
| 7 | 1235.2311754666669 | 5743.433436 |
| 12 | 1769.7884459666666 | 5045.982405333333 |
### Chart: PNPLA3
| Category | Cotrol | PCOS |
|---|---|---|
| 0 | 3.650824919135237 | 3.27026148262351 |
| 3 | 7.405502832514249 | 9.33378156311263 |
| 12 | 3.510478656343144 | 5.289306513757783 |G
O
### Chart: FTM2
| Category | Control | PCOS |
|---|---|---|
| 0 | 3.686732312 | 3.6843014733333335 |
| 3 | 9.506273631 | 18.300647976666667 |
| 7 | 12.045617303 | 27.023068679999998 |
| 12 | 14.994363158333334 | 27.82139374333333 |
### Chart: FITM2
| Category | Cotrol | PCOS |
|---|---|---|
| 0 | 2.9647538915168066 | 2.1439298871327868 |
| 3 | 8.50838425231066 | 11.687178484212737 |
| 12 | 4.831275818166227 | 7.562874316723556 |H
PCOS
CTL
Day

## Slide 8
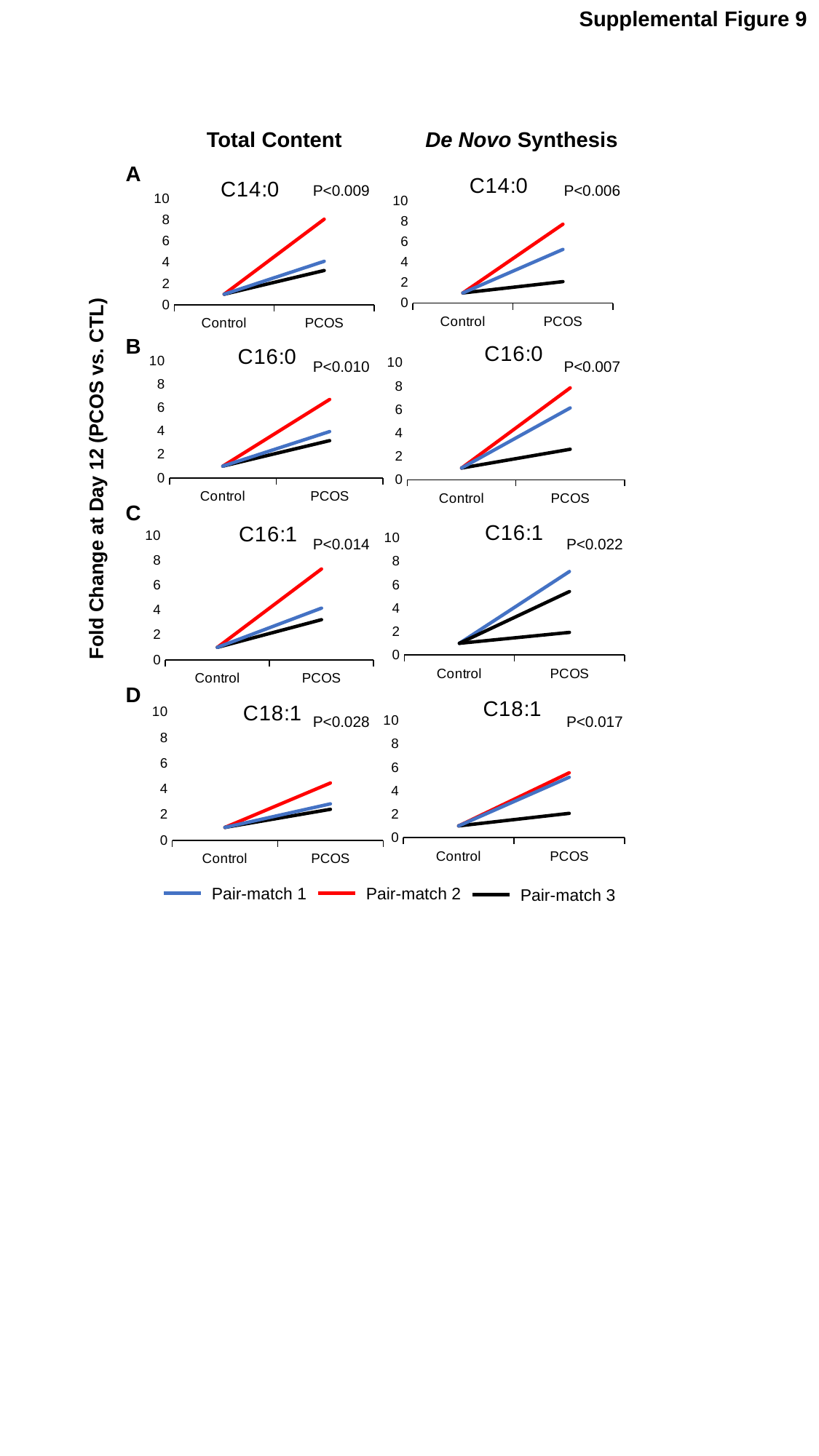

Supplemental Figure 9
Total Content
De Novo Synthesis
### Chart: C14:0
| Category | | | |
|---|---|---|---|
| Control | 1.0 | 1.0 | 1.0 |
| PCOS | 8.081245775545119 | 3.23627264776194 | 4.103081292553092 |A
### Chart: C14:0
| Category | | | |
|---|---|---|---|
| Control | 1.0 | 1.0 | 1.0 |
| PCOS | 7.701126002585715 | 2.100348429887202 | 5.244745934284786 |P<0.009
P<0.006
### Chart: C16:0
| Category | | | |
|---|---|---|---|
| Control | 1.0 | 1.0 | 1.0 |
| PCOS | 6.704324907128575 | 3.181414404156578 | 3.958460432753414 |
### Chart: C16:0
| Category | | | |
|---|---|---|---|
| Control | 1.0 | 1.0 | 1.0 |
| PCOS | 7.865638975631588 | 2.605934937251153 | 6.143694203489749 |B
P<0.010
P<0.007
Fold Change at Day 12 (PCOS vs. CTL)
### Chart: C16:1
| Category | | | |
|---|---|---|---|
| Control | 1.0 | 1.0 | 1.0 |
| PCOS | 7.300473813035837 | 3.222963087664613 | 4.151324839379072 |
### Chart: C16:1
| Category | | | |
|---|---|---|---|
| Control | 1.0 | 1.0 | 1.0 |
| PCOS | 7.149553624182027 | 1.931120305093628 | 5.43054199025366 |C
P<0.014
P<0.022
### Chart: C18:1
| Category | | | |
|---|---|---|---|
| Control | 1.0 | 1.0 | 1.0 |
| PCOS | 4.463948799361506 | 2.41427645743873 | 2.837687890849493 |
### Chart: C18:1
| Category | | | |
|---|---|---|---|
| Control | 1.0 | 1.0 | 1.0 |
| PCOS | 5.544268797777855 | 2.073917653255669 | 5.160218382718154 |D
P<0.028
P<0.017
Pair-match 1
Pair-match 2
Pair-match 3
